# Stereochemistry Controls Dihydrogen Bonding Strengths in Chiral Amine Boranes Adducts

**DOI:** 10.1002/anie.202213859

**Published:** 2022-11-27

**Authors:** Michael Kemper, Deborah A. Drost, Elric Engelage, Christian Merten

**Affiliations:** ^1^ Ruhr Universität Bochum Fakultät für Chemie und Biochemie Organische Chemie II Universitätsstraße 150 44801 Bochum Germany

**Keywords:** Hydrogen Bonding, Intermolecular Interactions, Stereochemistry, Thermochemistry, Vibrational Spectroscopy

## Abstract

The growing interest in exploiting novel concepts of non‐covalent interactions in catalysts and supramolecular chemistry made us revisit a special kind of hydrogen bonding: the dihydrogen bond (DHB), formed between a classical hydrogen bond donor and a hydridic hydrogen as acceptor. Herein, we investigate how the strength of the N−H^δ+^⋅⋅⋅^δ−^H−B interaction and hence the DHB‐driven self‐aggregation of amine‐borane adducts is governed by steric effects by comparing the structures and binding enthalpies of various chiral derivatives. For a diastereomeric pair of amine‐boranes prepared from a chiral secondary amine, we show that the stereochemistry at the nitrogen has significant influence on the interaction enthalpy. Based on this finding, N‐chiral amine boranes can be envisioned to become interesting building blocks in supramolecular chemistry to fine‐tune the formation dynamics of assemblies.

## Graphical abstract

How the strength of the N−H^δ+^⋅⋅⋅^δ^−H−B interaction and hence the DHB‐driven self‐aggregation of amine‐borane adducts is governed by steric effects has been investigated by comparing the structures and binding enthalpies of various chiral derivatives. For a diastereomeric pair of amine‐boranes prepared from a chiral secondary amine, it is shown that the stereochemistry at the nitrogen atom has a significant influence on the interaction enthalpy.

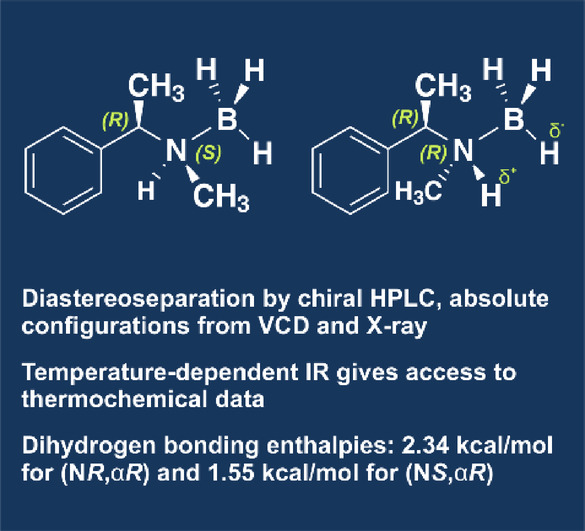

Non‐covalent interactions play important roles in various chemical systems. While there is a recent interest in employing novel concepts such as halogen,^[1]^ pnictogen^[2]^ and chalcogen^[3]^ bonding in catalysis and supramolecular chemistry, Coulomb and hydrogen bonding (HB) interactions are the most widely utilized interactions when it comes to directed design of functional molecules. A classical HB is formed between a proton donor X−H^δ+^ (with X being an electronegative element such as O or N) and the lone pair of another electronegative element :Y as acceptor.^[4]^ The interaction leads to an elongation of the X−H bond, which results in the well‐known red‐shift of the corresponding stretching vibrations. Apart from classical X−H^δ+^ structures also non‐conventional HBs are utilized in catalysis and ion‐recognition, for instance with C−H of triazoles as donor.^[5]^

Interestingly, a specific type of hydrogen bonding has not yet generated much attention in supramolecular chemistry and it is only little discussed in context of catalysis: the dihydrogen bond (DHB).^[6]^ It is formed between a conventional hydrogen bond donor X−H^δ+^ and a partial negatively charged hydrogen atom in ^δ−^H−Y, in which Y is less electronegative than H. Such proton‐hydride interaction can be found, for instance, in amine‐boranes (N−H^δ+^⋅⋅⋅^δ−^H−B). The first classification of DHBs was based on crystal structures, mostly of metal hydrides,[^6b^, ^7^] and most experimental data is only available for solid state structures. In amine‐boranes, the average distance of the H^δ+^⋅⋅⋅^δ−^H interaction is 1.97±0.2 Å and the DHB features binding angles N−H^δ+^⋅⋅⋅^δ−^H and H^δ+^⋅⋅⋅^δ−^H−B of about 150° and 120°, respectively.^[6b]^ The computed structure of the prototypical dimer (H_3_N−BH_3_)_2_ is a *C*_2*h*_‐symmetric head‐to‐tail complex with two sets of bifurcated DHBs.^[8]^ Recent applications of amine‐borane adducts and thus systems,^[9]^ in which dihydrogen bonding can occur, often focus on application in hydroboration but they recently also gained attention in the polymer and material sciences as dehydro‐coupling,^[10]^ the simultaneous release of H_2_ and the formation of a new dative B−N bond, gives access to poly(aminoboranes) and novel hydrogen storage materials.

Studies on chiral amine‐boranes and their dihydrogen bonding behavior, which could potentially become interesting for the design of functional supramolecular structures or asymmetric catalysts, are particularly sparse. In fact, to the best of our knowledge, the first chiral amine‐borane studied with focus on its dihydrogen bonding was α‐methylbenzyl amine borane (**MBA‐BH_3_**, Scheme 1).^[11]^ **MBA‐BH_3_** was investigated using vibrational circular dichroism (VCD) spectroscopy, the chiral version of infrared spectroscopy, which is particularly known as a reliable tool for the determination of absolute configurations.^[12]^ In recent years, VCD spectroscopy has also been used in our group to investigate intermolecular interactions such as self‐aggregation and solvation, for the characterization of supramolecular assemblies and active species in asymmetric catalysis.^[13]^ The VCD spectra of **MBA‐BH_3_** were found to be sensitive to DHB‐driven dimerization and the structure of the dimeric complex could unambiguously be determined in an apolar solvent by comparison with computed spectra.^[11]^ We subsequently investigated the bis(α‐methylbenzyl)amine‐BH_3_ adduct (**Bis(MBA)‐BH_3_**), which we found to interact through a conventional hydrogen bond with a single solvent molecule in ACN solution without forming dimers. In weakly polar CDCl_3_, monomer and dimer could not be distinguished based on their VCD spectra.^[14]^

**Scheme 1:**
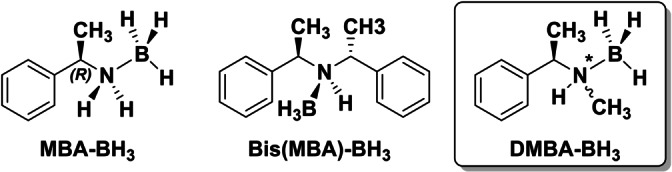
Structures of previously investigated amine‐boranes **MBA‐BH_3_** and **Bis(MBA‐BH_3_)** and the target molecule of this study, **DMBA‐BH_3_**. Note that DMBA‐BH_3_ exists in two diastereomeric forms as the nitrogen becomes a stereocenter.

The results of the two studies revealed notably different aggregation behavior of **MBA‐BH_3_** and **Bis(MBA)‐BH_3_**. While the primary amine derivative forms head‐to‐tail dimers, the secondary amine borane crystallizes in a helical structure with an open chain‐like DHB network. Steric constraints thus obviously determine the dimer topology, but do not hinder the amine boranes from self‐aggregating.

In this study, we investigated borane adducts of the sterically less hindered secondary amine N,α‐dimethylbenzyl amine (DMBA), which we considered as ideal intermediate between the two aforementioned cases to further investigate the role of chirality in the formation of dihydrogen bonded aggregates. Besides the carbon‐based stereocenter, the non‐symmetric substitution renders the nitrogen atom pro‐chiral. Hence, if the resulting mixture of the diastereomers (N*R*,α*R*)‐ and (N*S*,α*R*)*‐* **DMBA‐BH_3_** (and analogously for (α*S*)*‐*DMBA as the starting amine) could be resolved into its enantiomers, the N‐stereocenter could become another handle to control the strength of the dihydrogen bonding interaction. To our delight, the ^1^H NMR spectrum of **DMBA‐BH_3_** indeed confirmed the presence of two species (cf. Figure S24/S25): Two clearly separated multiplets arising from the methylene protons integrate to a ratio of 70 : 30 between the two epimers. More importantly, we subsequently succeeded in separating the epimers of **DMBA‐BH_3_** using chiral HPLC (YMC Chiral ART Cellulose‐SC, cyclohexane/*i*PrOH=9/1; cf. Supporting Information for more details), which confirms that the nitrogen atom becomes a stable stereocenter. The short retention times of 6 min for the major and 7.9 min for the minor epimer allowed us to resolve reasonable amounts for further studies already on an analytical column.

The absolute configurations of the resolved epimers were first established by means of VCD spectroscopy. To this end, we recorded the IR and VCD spectra of both pairs of enantiomers in a polar solvent (ACN‐d_3_) in order to avoid DHB‐driven self‐aggregation. The corresponding computational analysis of the spectra, that means, the conformational analysis and spectra predictions, were carried out at the B3LYP/6‐31G+(2d,p)/IEFPCM(ACN) level of theory. The direct comparison of the experimental spectra of the major species and the computed spectra of the (N*R*,α*R*)‐epimer (Figure 1) reveals a very good resemblance of basically all spectral features. Likewise, the computed spectral signatures of the (N*S*,α*R*)‐stereoisomer match very well with the minor species’ experimental data. Note that explicit consideration of N−H⋅⋅⋅NCCD_3_ hydrogen bonds led to a worsening of the match (Figure S17). The first eluting, major isomer can thus be identified as (N*R*,α*R*)*‐* **DMBA‐BH_3_**, while the minor product possesses (N*S*,α*R*)‐chirality.

**Figure 1:**
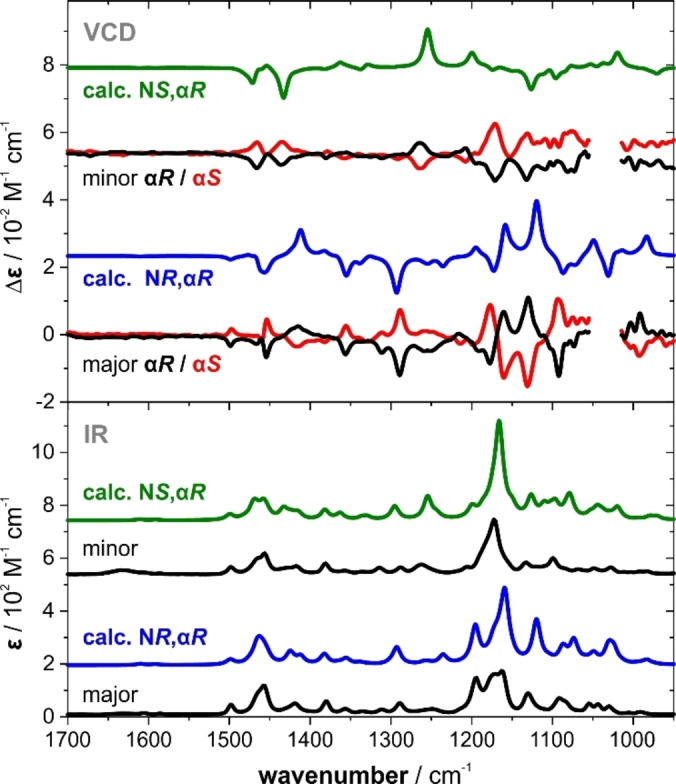
Comparison of the experimental IR and VCD spectra of both enantiomers of each epimer of **DMBA‐BH_3_** with the computed spectra of the (N*R*,α*R*)‐ and (N*S*,α*R*)‐epimers. For better comparison, the intensities of the experimental IR and the computed VCD of the (N*S*,α*R*)‐epimer are scaled by a factor of 2 and 0.5, respectively.

The solid‐state structure of (N*R*,α*R*)*‐* **DMBA‐BH_3_** could be determined from a single crystal grown from toluene solution.^[15]^ It shows isolated C_2_‐symmetric head‐to‐tail dimer structures with a bifurcated DHB topology (Figure 2). This is in contrast to **MBA‐BH_3_**, for which aggregation in a head‐to‐tail structure still allows the formation of an infinite DHB network within the crystal.^[11]^ The additional methyl group of DMBA thus effectively prevents the formation of larger aggregates, without forcing the superstructure into open‐chain interactions as in the case of **Bis(MBA)‐BH_3_**.^[14]^ Unfortunately, a direct comparison with the (N*S*,α*R*)**‐DMBA‐BH_3_** epimer was not possible as we did not obtain crystals suitable for X‐ray crystallography but only oily samples.

**Figure 2:**
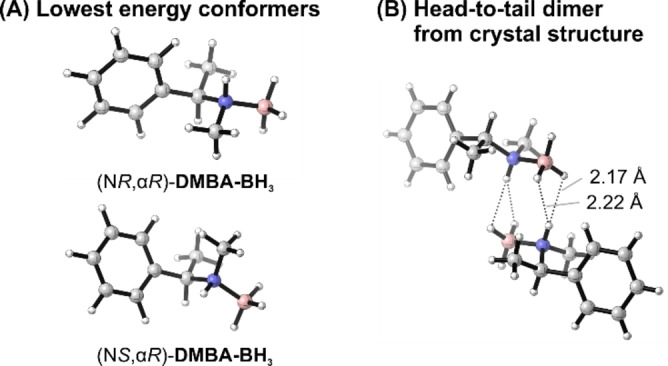
A) Computed lowest energy conformers of (N*R*,α*R*)‐ and (N*S*,α*R*)*‐* **DMBA‐BH_3_**. B) Crystal structure of (N*R*,α*R*)*‐* **DMBA‐BH_3_**.

In order to gain further information on the DHB‐interactions of both epimers and especially insights into their energetics, we characterized the solution phase structures of the amine‐boranes in a weakly polar solvent (DCM‐d_2_) by IR spectroscopy. For both epimers, concentration‐dependent IR measurements reveal characteristic changes in the N−H stretching region (3300–3100 cm^−1^, Figure S1): With increasing concentration of **DMBA‐BH_3_**, a band at ≈3270 cm^−1^ decreases in relative intensity, while a band at 3200 cm^−1^ grows. The higher‐frequency band can be assigned to the N−H stretching mode of monomeric **DMBA‐BH_3_** and the red‐shifted band originates from N−H bonds involved in a DHB.^[14]^ Interestingly, for spectra recorded at the same concentration, the ratio of the (integrated) intensities of these bands is different for the two epimers (cf. Figure 3, 20 °C measurements). This observation already suggests that the dimer of (N*R*,α*R*)**‐DMBA‐BH_3_** is energetically more favored than the (N*S*,α*R*)‐dimer. In order to experimentally determine the formation enthalpy Δ*H*_DHB_ of the dihydrogen bonded clusters, temperature‐dependent IR measurements were carried out. With decreasing temperature, the formation of dimers could again be monitored as an increase in the intensity of the ≈3200 cm^−1^ band (Figure 3). The analysis of the spectra by means of a van't Hoff plot gave Δ*H*_DHB_ of −2.34 kcal mol^−1^ for (N*R*,α*R*)**‐DMBA‐BH_3_** and −1.55 kcal mol^−1^ for the (N*S*,α*R*)‐epimer (cf. Supporting Information, Figure S5–S10). A similar analysis of the as‐prepared mixture of epimers (approximately 7 : 3 ratio) gave a Δ*H*_DHB_ of −2.14 kcal mol^−1^. Interestingly, this Δ*H*_DHB_ of the epimer mixture is almost identical to the contribution‐weighted average of the Δ*H*_DHB_ values of the enantiopure compounds (70 % of −2.34 kcal mol^−1^ and 30 % of −1.55 kcal mol^−1^ equal −2.1 kcal mol^−1^). While this may hint at a preferential aggregation of the same stereoisomers over the formation of mixed aggregates, the IR and ^1^H NMR spectra of the diastereomeric mixture were simply superpositions of the epimers’ spectra. Thus, an unequivocal distinction between homo‐ and heterodimeric species is not possible based on the available spectroscopic data.

**Figure 3:**
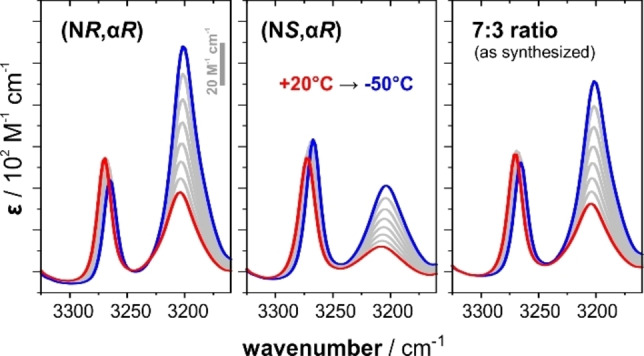
Variable‐temperature IR spectra of (N*R*,α*R*)‐ and (N*S*,α*R*)*‐* **DMBA‐BH_3_** and for the mixture of epimer obtained from synthesis. Measurements were done in DCM‐d_2_ and recorded in steps of 10 °C from +20 °C to −50 °C.

As we have previously found the VCD spectra of **MBA‐BH_3_** in weakly polar CDCl_3_ to be characteristic for the DHB topology of the amine borane dimer in solution phase,^[11]^ we also recorded the VCD spectra of (N*R*,α*R*)**‐DMBA‐BH_3_** in DCM‐d_2_. Interestingly, comparing the VCD spectra recorded at 0.54 M and 1.08 M did not show any notable difference that could be used as marker band for dimer formation in the VCD fingerprint range (Figure S18). Likewise, lowering the temperature to −50 °C also did not lead to changes in the VCD fingerprint region of (N*S*,α*R*)**‐DMBA‐BH_3_** (Figure S19). VCD spectra calculations later confirmed that such differences are also not to be expected. Similar to the case of **Bis(MBA)‐BH_3_**,^[14]^ only the IR bands of the N−H and B−H stretching modes are sensitive to DHB interactions.

Comparing the experimental Δ*H*_DHB_ values, it must be highlighted that the difference between the DHB‐enthalpies of the two epimers is surprisingly large considering the structural similarity of the epimers. Consequently, we also determined the Δ*H*_DHB_ of other amine‐boranes (Table 1, Figure S11–S16). The primary amine derivative **MBA‐BH_3_** was found to have a larger Δ*H*_DHB_ than **DMBA‐BH_3_** (−3.16 kcal mol^−1^), while the sterically least demanding benzylamine‐borane (**BzA‐BH_3_**) is found in the same range (−1.87 kcal mol^−1^) as **DMBA‐BH_3_**. Expectedly, the sterically more demanding **Bis(MBA)‐BH_3_** features the lowest Δ*H*_DHB_ (−0.78 kcal mol^−1^). Note that the frequency difference between the bands of the free N−H and the hydrogen‐bonded N−H stretching vibrations are often considered a measure for and to be proportional to the hydrogen‐bonding strength.^[16]^ In the present case, however, they follow a different order than the enthalpies: It is the smallest for the primary amines, followed by **Bis(MBA)‐BH_3_** and biggest for the epimers of **DMBA‐BH_3_** (Table 1). Furthermore, for (N*R*,α*R*)‐ and (N*S*,α*R*)*‐* **DMBA‐BH_3_** the same shift Δν of 65 cm^−1^ is observed although experimental Δ*H*_DHB_ values differ by about 0.7 kcal mol^−1^. Accordingly, the shift of the N−H stretching mode and the experimentally determined enthalpies obviously do not correlate for the investigated series of amine‐boranes.

**Table 1:** Comparison of the aggregation enthalpies of DMBA‐BH_3_ with those of other amine‐boranes (relative zero‐point corrected energies Δ*E*_ZPC_ and enthalpies Δ*H* are given in kcal mol^−1^; HT=percentage contribution of head‐to‐tail dimers). Computational level: B3LYP/6‐311++G(2d,p)/IEFPCM(DCM).

	experiment^[a]^		computation		
	Δ*H*_DHB_	Δν^[b]^	Δ*E*_ZPC_	Δ*H*	HT
(N*R*,α*R*)*‐* **DMBA‐BH_3_**	−2.34	65	−2.70	−2.07	86
(N*S*,α*R*)*‐* **DMBA‐BH_3_**	−1.55	65	−1.89	−1.27	69
7 : 3 mixture	−2.14	65			
**MBA‐BH_3_**	−3.16	47	−2.96	−2.35	99
**BzA‐BH_3_**	−1.87	44	−2.92	−2.41	97
**Bis(MBA)‐BH_3_**	−0.78	55	−1.08	−0.35	84

[a] experimental data can be found in the Supporting Information; [b] Δν=ν_(freeNH)_–ν_(HbondedNH)_ as determined at 20 °C.

Open‐chain dimers feature single DHBs between the amine‐borane monomers, while the ideal head‐to‐tail dimer is typically characterized by two bifurcated DHBs. One may thus argue that a lesser extent of head‐to‐tail dimers contributing to the conformational equilibrium may lead to lower binding enthalpies due to fewer attractive interactions per dimer unit. While the available crystal structure data for **MBA‐BH_3_**, (N*R*,α*R*)*‐* **DMBA‐BH_3_** and **Bis(MBA)‐BH_3_** seem to somewhat confirm this trend, we computed the conformational equilibria of the dimers of all five investigated amine‐boranes to obtain a more complete picture. To this end, we built and optimized head‐to‐tail and open‐chain dimeric structures of both epimers of **DMBA‐BH_3_** and of **BzA‐BH_3_**. In addition, we retrieved the structures from our previous studies[^11^, ^14^] and re‐optimized them, so that all calculations were eventually done at the same level of theory. The computed data, i.e. the dimerization energies and the percentage contribution of head‐to‐tail dimers to the conformational equilibrium (based on Δ*E*_ZPC_), are summarized in Table 1.

Examining first the computed dimerization energies (cf. Supporting Information for a description of the computational procedure), it can be noted that they follow mostly the experimentally determined order. Solely the experimentally observed clear difference between the primary amine boranes **MBA‐BH_3_** and **BzA‐BH_3_** is not reflected in the computed data, which predicts them to have basically identical tendencies to form dimers. This similarity cannot be lifted by changing the implicit solvation model to SMD^[17]^ (which generally decreases the dimerization energies) or by considering dispersion interactions by means of the GD3BJ correction^[18]^ (which generally leads to drastic stabilization of all dimers; cf. Tables S13 and S18). It may thus suggest the formation of potentially larger aggregates in solution phase for both primary amine boranes.

Focusing on structural aspects of the dimers, we find head‐to‐tail structures to dominate the conformational distributions in all five cases. In order to gain a more detailed picture of the different intermolecular interactions determining the dimerization enthalpies, we examined the structures of the lowest energy head‐to‐tail conformations of each amine‐borane in more detail (Figure 4). The two hydrogen bonding distances in the bifurcated dimers of the primary amine boranes **MBA‐BH_3_** and **BzA‐BH_3_** and of the major (N*R*,α*R*)‐isomer of **DMBA‐BH_3_** were found to be very similar: The two H^δ+^⋅⋅⋅^δ−^H hydrogen bond lengths differ only by 0.07–0.12 Å. Likewise, the lengths of the DHBs are quite comparable among the three amine‐boranes. In contrast, (N*S*,α*R*)*‐* **DMBA‐BH_3_** and **Bis(MBA)‐BH_3_** (shown in the Supporting Information only; Figure S25) deviate drastically from the ideal bifurcated head‐to‐tail geometry with the two H^δ+^⋅⋅⋅^δ−^H distances differing by 0.59 and 0.78 Å, respectively. For **Bis(MBA)‐BH_3_**, this elongation of one bond results in a displacement of the monomers with respect to each other, while for (N*S*,α*R*)*‐* **DMBA‐BH_3_** the N−B axes are not parallel anymore. The first three amine‐boranes can thus be considered to possess two bifurcated DHBs (one on each head‐tail side), while the latter two are better described as head‐to‐tail dimers featuring only a single DHB. This observation suggests a correlation between the dihydrogen bonding topology and dimerization enthalpy.

**Figure 4:**
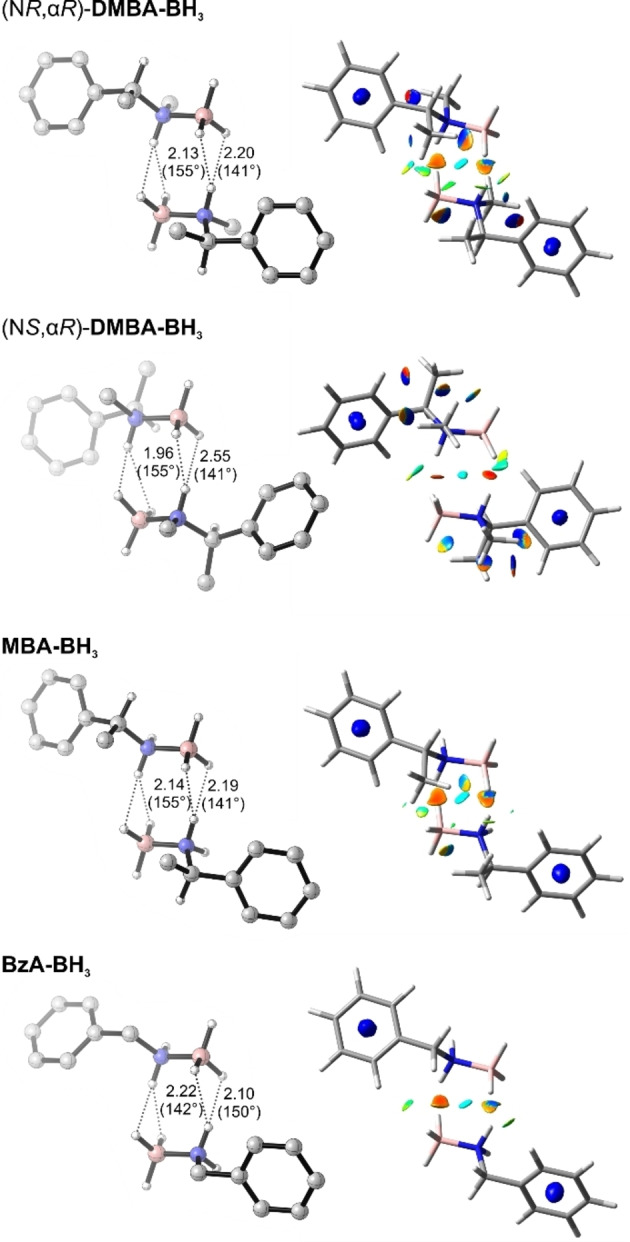
Structures of the lowest energy head‐to‐tail dimers of the investigated amine‐boranes (H^δ+^⋅⋅⋅^δ−^H distances in Å, N− H^δ+^⋅⋅⋅^δ−^HB angles in degree) and NCI plots. The NCI surfaces are plotted on the 0.3 au reduced density gradient isosurface and colored from red (attractive interactions) to blue (repulsive interactions) in a range from −2.0 to 2.0. The green color indicates vdW interactions. Non‐relevant hydrogen atoms are omitted for clarity. Additional views are provided in the Supporting Information (Figure S21–S24).

A further qualitative evaluation of the intermolecular interactions can be obtained from a visualization of the intra‐ and intermolecular non‐covalent contacts by depicting the reduced density gradient in regions of low electron density (NCIPLOT).^[19]^ In these representations (Figure 4), strongly attractive and repulsive interactions are shown as red and blue regions (isosurfaces), respectively. Weak non‐covalent interactions, that are usually considered to arise from attractive London dispersion, are shown in green. Focussing first on the NCI plot of **BzA‐BH_3_** the red spots in the DHB contact plane represent the driving interaction between the two monomers. A second interaction, that is shown as green spots, is present between a B−H bond and the C_ortho_‐H of the respective other monomer. In addition to some intramolecular contacts between the α‐methyl and the BH_3_‐groups and the intermolecular B−H⋅⋅⋅H−C_ortho_ contacts, the NCI plot of **MBA‐BH_3_** also shows intermolecular contacts of the same B−H bond with the α‐methyl group of the respective other monomer units. Hence, although the binding geometry is basically identical for these two dimers, London dispersion is likely to become the dominant interaction leading to a stronger stabilization of the **MBA‐BH_3_** compared to those of **BzA‐BH_3_** when the size of the aggregates grows beyond dimers. Likewise, examination of the **DMBA‐BH_3_** isomers reveals the same types of contacts to be responsible for the energetic differences. In fact, it can immediately be noted that the major (N*R*,α*R*)‐isomer possesses more intermolecular contacts than the weaker binding (N*S*,α*R*)‐isomer. While (N*S*,α*R*)*‐* **DMBA‐BH_3_** only shows the B−H⋅⋅⋅H−C_ortho_ interaction, the α‐methyl groups are again also involved in case of the major isomer (N*R*,α*R*)*‐* **DMBA‐BH_3_**.

In summary we could show that there are two closely related factors that determine the DHB interaction energies of the chiral amine boranes: The DHB topology itself (single linear vs. bifurcated interactions) and intermolecular dispersive contacts. Both factors relate to the steric situation around the N−B units and, as shown for **DMBA‐BH_3_**, the configuration of the N‐stereogenic center can be used to control these interactions. Perspectively, the N‐stereogenic center could thus provide a handle to balance such aggregation enthalpies and to utilize DHBs for the controlled formation of larger supramolecular aggregates in the future.

## Conflict of interest

The authors declare no conflict of interest.

## Supporting information

As a service to our authors and readers, this journal provides supporting information supplied by the authors. Such materials are peer reviewed and may be re‐organized for online delivery, but are not copy‐edited or typeset. Technical support issues arising from supporting information (other than missing files) should be addressed to the authors.

Supporting Information

Supporting Information

## Data Availability

The data that support the findings of this study are available in the Supporting Information of this article.
