# Supplementary material for: Stereochemistry Controls Dihydrogen Bonding Strengths in Chiral Amine Boranes Adducts
Source: Angew Chem Int Ed Engl. 2022 Nov 27;61(52):e202213859. doi: 10.1002/anie.202213859 (PMC10099978; doi:10.1002/anie.202213859)
Supplement: Supplementary file 1 — Supporting Information [file ANIE-61-0-s001.pdf]

Angewandte Chemie
Eine Zeitschrift der Gesellschaft Deutscher Chemiker

Supporting Information

Stereochemistry Controls Dihydrogen Bonding Strengths in Chiral Amine Boranes Adducts

*M. Kemper, D. A. Drost, E. Engelage, C. Merten**

1. Preparation and enantioseparation of DMBA-BH_3 2
2. Computational details 4
3. Vibrational spectroscopy 4
4. Thermochemistry 5
5. Additional spectra 11
6. NCI plots 13
8. Crystallography 26
9. NMR spectra 29
10. References 33

1. Preparation and enantioseparation of DMBA-BH3

Synthetic procedure

Chiral amines and BH_3-DMS were acquired from Merck an used without further purification.

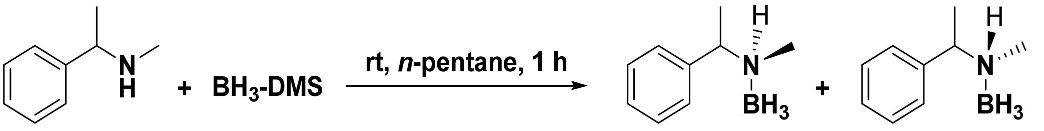

(N*R*)-**DMBA-BH3** (N*S*)-**DMBA-BH3**

In a round-bottom flask enantiopure **DMBA** (93.3 mg, 0.10 mL, 0.69 mmol, 1.0 eq.) was dissolved in *n*-pentane and BH_3-DMS (57.7 mg, 0.07 mL, 0.76 mmol, 1.1 eq.) was added dropwise. The mixture was stirred at room temperature for 1 h during which a colourless solid precipitated. The solvent was removed under reduced pressure at room temperature and the resulting colourless solid was purified by flash-chromatography on silica (eluent: dichloromethane). The product was obtained as a diastereomeric mixture with a yield of 99 % (101.4 mg, 0.68 mmol).

High Performance Liquid Chromatography

The diastereomeric mixtures of (N*R*)-/(N*S*)-**DMBA-BH**$_3$ were separated on a Shimadzu HPLC system equipped with autosampler and fraction collector using a YMC CHIRAL ART Cellulose SC S-5 µm column at a flowrate of 1 mL·min^{-1}. A mixture of cyclohexane and 2-propanol was used as eluent (ratio = 90:10) and as solvent to prepare sample solutions of 20 mg·mL^{-1}. Injection volumes were kept below 100 µL to prevent excessive peak broadening. Retention times were 5.99 min and 7.85 min respectively. The ratio of peak areas was determined as 77 % to 23 %. For the minor species, decomposition was observed when heated above room temperature.

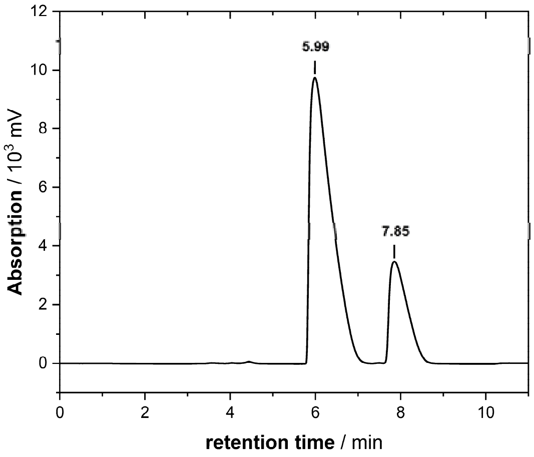

Summary of analytical data

^{1}H-NMR (N*R*,α*R*)/(N*S*,α*S*)-**DMBA-BH₃** (200 MHz, ACN-*d₃*): δ/ppm = 7.49-7.25 (5H, m, C*H*-ar), 4.47 (1H, s_{br}, N*H*), 3.78-3.63 (1H, *m*, C*H*), 2.18 (3H, d, $^3J_{H-H}$ = 5.67 Hz, N-C*H*₃), 1.75 (1H, s_{br}, B*H*), 1.57 (3H, d, $^3J_{H-H}$ = 6.89 Hz, C*H*₃), 1.40-1.01 (1H, m, B*H*), 0.79 (1H, s_{br}, B*H*).

^{1}H-NMR (N*S*,α*R*)/(N*R*,α*S*)-**DMBA-BH₃** (200 MHz, ACN-*d₃*): δ/ppm = 7.41-7.31 (5H, m, C*H*-ar), 4.57 (1H, s_{br}, N*H*), 4.14-4.05 (1H, m, C*H*), 2.24 (3H, d, $^3J_{H-H}$ = 5.78 Hz, N-C*H*₃), 1.50 (1H, m, B*H*), 1.52 (3H, d, $^3J_{H-H}$ = 7.06 Hz, C*H*₃), 1.27 (1H, m, B*H*), .094 (1H, s_{br}, B*H*).

^{13}C-NMR (N*R*,α*R*)/(N*S*,α*S*)-**DMBA-BH₃** (50 MHz, ACN-*d₃*): δ/ppm = 141.61, 129.64, 129.11, 128.64, 66.79, 41.82, 20.78.

^{13}C-NMR (N*S*,α*R*)/(N*R*,α*S*)-**DMBA-BH₃** (50 MHz, ACN-*d₃*): δ/ppm = 140.44, 129.48, 129.33, 129.25, 63.22, 37.08, 14.83.

^{11}B-NMR (N*R*,α*R*)/(N*S*,α*S*)-**DMBA-BH₃** (128 MHz, ACN-*d₃*): δ/ppm = -15.46 (q, $^1J_{B-H}$ = 96.6 Hz).

^{11}B-NMR (N*S*,α*R*)/(N*R*,α*S*)-**DMBA-BH₃** (128 MHz, ACN-*d₃*): δ/ppm = -15.63 (q, $^1J_{B-H}$ = 103.5 Hz)

MS (ESI-pos.) (N*R*,α*R*)/(N*S*,α*S*)--**DMBA-BH₃**: *m*/*z*: calcd. for $[M+H]^+$: 150.15. Found: 150.1

calcd. for $[M-BH_3+H]^+$: 136.11. Found 136.1

MS (ESI-pos.) (N*S*,α*R*)/(N*R*,α*S*)-**DMBA-BH₃**: *m*/*z*: calcd. for $[M-BH_3+H]^+$: 136.11. Found 136.1

2. Computational details

General

Conformational searches for all investigated compounds were carried out systematically by preparing individual starting structures for unique combinations of torsional angles (threefold-rotation). All calculations were carried out using Gaussian 09 Rev. E.01[1] employing the B3LYP/6-31+G(2d,p) and B3LYP/6-311++G(2d,p) level of DFT. Mostly the IEF-PCM solvation model for ACN and DCM was applied. For selected cases, also SMD was used (see text). IR and VCD spectra were simulated by assigning a uniform Lorentzian band shape of 6 cm^{-1} half-width at half-height to the computed dipole and rotational strengths. The vibrational spectra presented in the main text are scaled with a frequency scaling factor σ of 0.98. Relative zero-point energy corrected energies, ΔE_{ZPC}, were used to determine Boltzmann weights.

Dimerization enthalphies

Dimerization energies were calculated based on the optimized structures of the dimers and the incorporated monomers by also taking into account the Boltzmann weight χ_{dimer} of the dimer:

$$\Delta E_{DHB} = \sum \chi_{dimer} \cdot (E_{dimer} - E_{monomer1} - E_{monomer2})$$

Note that this procedure does not consider any basis set superposition error (BSSE). Evaluation of this additional step was left out as a few test calculations showed that the magnitude of the effect is small and in the range of the energetic cost associated with conformational changes (which are also not explicitly considered either).

3. Vibrational spectroscopy

Temperature dependent IR measurements were carried out using a JANIS ST-100 cryostat, in which samples were held in a vacuum-tight, sealed BaF_2 cell with 100 µm path length. Cooling was achieved by a constant flow of liquid nitrogen, while temperature regulation was achieved with a Lakeshore temperature controller. After a change in measurement temperature, sample cell and cryostat were allowed to reach thermal equilibrium. IR spectra were recorded with 4 cm^{-1} spectral resolution by accumulating for 32 scans and repeated 10 times in time intervals of 1 min.

VCD spectra were recorded on a Bruker Vertex FT-IR spectrometer equipped with a PMA 50 module for VCD measurements. The sample was held in a transmission cell with BaF_2 windows and 100 µm path length. Concentration are given in the main text. Spectra were recorded at room temperature with 4 cm^{-1} spectral resolution by accumulating 32 scans for the IR and ~16000 scans (4 hours accumulation time) for VCD. Baseline correction of the VCD spectra was done by subtraction of the spectra of the solvent or the racemic mixture recorded under identical conditions.

4. Thermochemistry

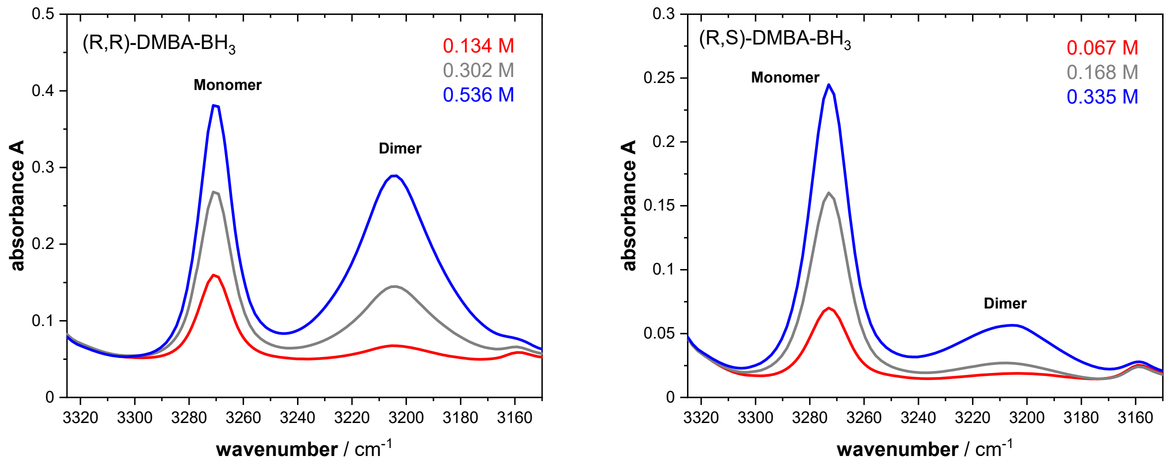

Fig S1. Concentration dependence of the IR spectra of the two epimers of DMBA-BH_3 in the N-H stretching mode region.

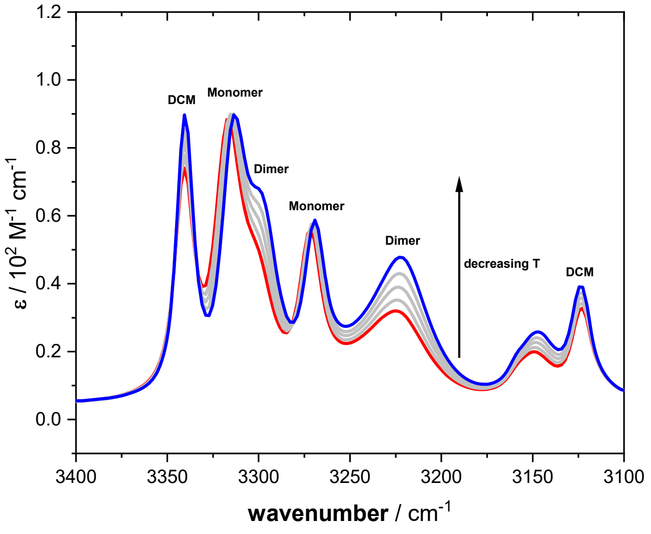

Fig. S2. Temperature dependent IR spectra of **MBA-BH₃** in the range of 3400 to 3100 cm^{-1}. Solvent = DCM-d_2, path length = 100 µm, c = 0.296 mol·L^{-1}, temperature range = 293.15 - 253.15 K.

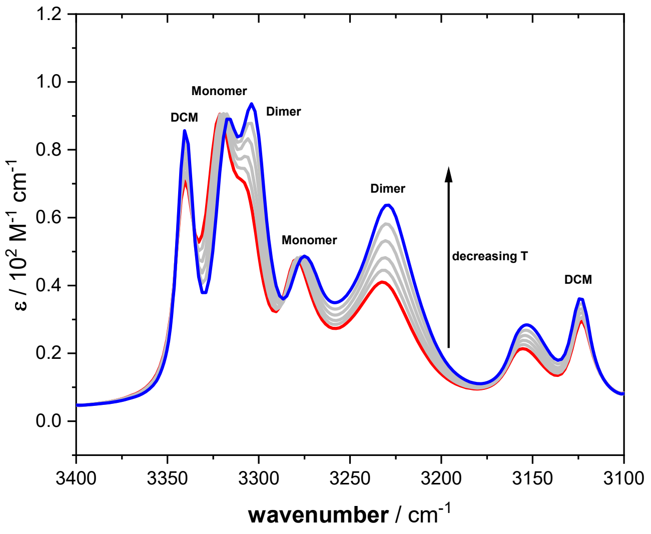

Fig. S3. Temperature dependent IR spectra of **BzA-BH$_3$** in the range of 3400 to 3100 cm^{-1}. Solvent = DCM-d_2, path length = 100 µm, c = 0.370 mol·L^{-1}, temperature range = 293.15 - 243.15 K.

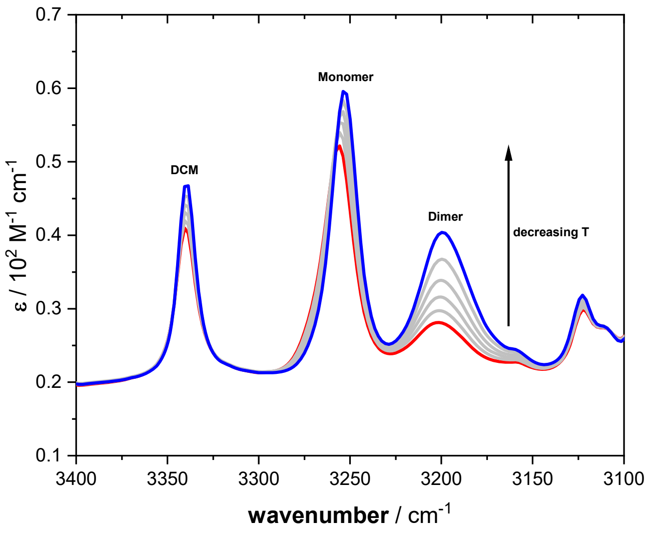

Fig. S4. Temperature dependent IR spectra of **Bis(MBA)-BH$_3$** in the range of 3400 to 3100 cm^{-1}. Solvent = DCM-d_2, path length = 100 µm, c = 1.78 mol·L^{-1}, temperature range = 293.15 - 243.15 K.

For the determination of the equilibrium constant K needed for the van't Hoff plot, the mole fractions of monomer and dimer at a given temperature of the VT-IR series were determined based on the integral intensities of the respective monomer bands in conjunction with the concentration dependent IR (VC-IR) measurements. To this end, the monomer bands of both VT-IR anc VC-IR series were integrated by fitting Lorentzian band shapes to the experimental data. To account for an increase in integral intensity due to increased density upon cooling of the sample, integral monomer intensities were corrected using the integral solvent intensities at the same temperature. The integral intensities (in km mol^{-1}) of a VC-IR series at 293 K were then plotted against the concentration (in mol L^{-1}). The intensity of the monomer band at infinite dilution (i.e. no dimer formation) can be approximated from this plot as the intercept b of a linear regression (y = mx + b) and the fraction of monomer $x_{M,293K}$ at a given concentration c1 and 293 K determined as:

$$x_{M,293K} = \frac{I_{Mc1,293K}}{b}$$

The solvent signal corrected integral monomer intensity at infinite dilution $I_{M,inf}$ of the VT-IR measurements is then determined as the fraction of the solvent corrected integral monomer intensity I_M and $x_{M,293K}$ according to:

$$I_{M,inf} = \frac{I_M}{x_{M,293K}}$$

The mole fraction of the monomer x_M at a given temperature is calculated as the fraction of the integral monomer intensity I_M and the monomer intensity at infinite dilution $I_{M,inf}$:

$$x_M = \frac{I_M}{I_{M,inf}}$$

The dimer mole fraction x_D is subsequently calculated from x_M as:

$$x_D = \frac{1 - x_M}{2}$$

The equilibrium constant K at a given temperature was then calculated from the monomer and dimer molefractions x_M and x_D according to:

$$K = \frac{x_D}{x_M x_M}$$

The slope m of a linear regression of the corresponding van't Hoff plot (ln(K) against T^{-1}) gives the dimerization enthalpy ΔH as:

$$\Delta H = -mR$$

With R being the ideal gas constant (8.314 J mol^{-1} K^{-1}). The plots of the VC-IR series and the corresponding van't Hoff plots are depicted below. The respective intercepts of the concentration dependence plots and the slopes of the van't Hoff plots from which the dimerization enthalpies were calculated are given.

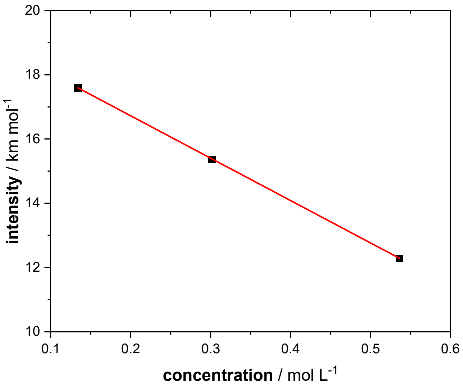

Fig. S5. (N*R*,α*R*)-DMBA-BH3 concentration dependence: Linear fit of integral intensity of monomer signal ~3270 cm^{-1} in relation to sample concentration of (N*R*,α*R*)-**DMBA-BH3**; m = -13.19, b = 19.36, R^2 = 1.

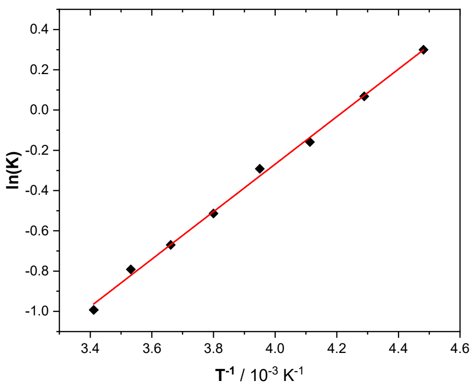

Fig. S6. (N*R*,α*R*)-DMBA-BH3 van't Hoff plot: Linear fit of experimental ln(K) values of (N*R*,α*R*)-**DMBA-BH3** against corresponding T^{-1} values. The slope was determined to be $-\Delta H \cdot R^{-1}$ = 1179.49 which corresponds to an enthalpy ΔH of -9.81 kJ·mol^{-1} or -2.34 kcal·mol^{-1}, R^2 = 0.997.

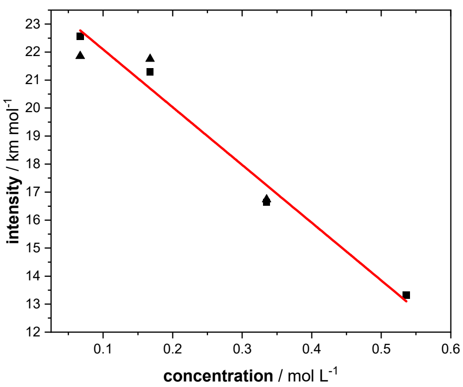

Fig. S7. (N*S*,α*R*)-DMBA-BH3 concentration dependence: Linear fit of integral intensity of monomer signal ~3270 cm^{-1} in relation to sample concentration of (N*S*,α*R*)-**DMBA-BH3**; m = -20.62, b = 24.16, R^2 = 0.985.

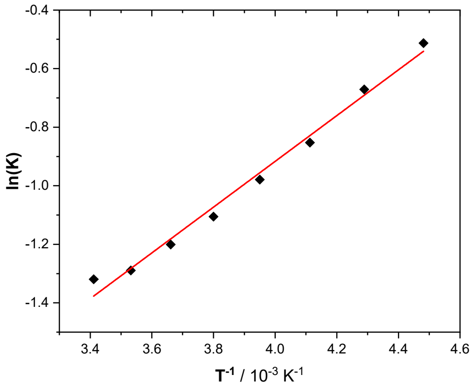

Fig. S8. **(N*S*,α*R*)-DMBA-BH3** van't Hoff plot: Linear fit of experimental ln(K) values of (N*S*,α*R*)-**DMBA-BH3** against corresponding T^{-1} values. The slope was determined to be $-\Delta H \cdot R^{-1}$ = 781.40 which corresponds to an enthalpy ΔH of -6.50 kJ·mol^{-1} or -1.55 kcal·mol^{-1}, R^2 = 0.988.

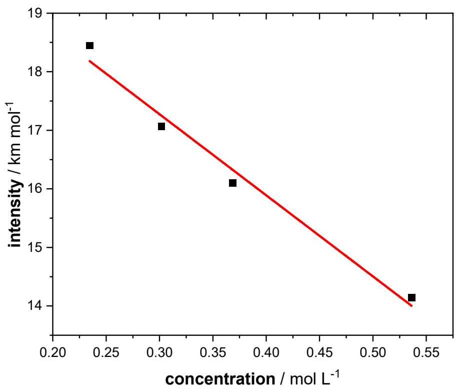

Fig. S9. Concentration dependence of **mixture of DMBA-BH$_3$ isomers**: Linear fit of integral intensity of monomer signal ~3270 cm^{-1} in relation to sample concentration of mixture of (N*R*,α*R*)-**DMBA-BH$_3$** and (N*S*,α*R*)-**DMBA-BH$_3$**; m = -13.85, b = 21.43, R^2 = 0.973.

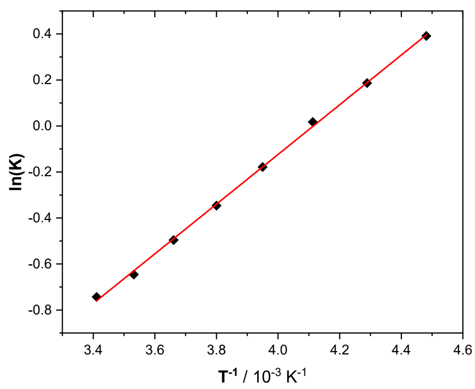

Fig. S10. van't Hoff plot of **mixture of DMBA-BH$_3$ isomers**: Linear fit of experimental ln(K) values of mixture of (N*R*,α*R*)**-DMBA-BH$_3$** and (N*S*,α*R*)**-DMBA-BH$_3$** against corresponding T^{-1} values. The slope was determined to be $-\Delta H \cdot R^{-1}$ = 1080.10 which corresponds to an enthalpy ΔH of -8.98 kJ·mol^{-1} or -2.14 kcal·mol^{-1}, R^2 = 0.999.

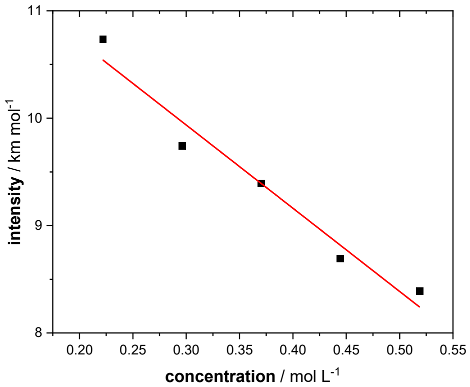

Fig. S11. MBA-BH$_3$ concentration dependence: Linear fit of integral intensity of monomer signal at ~3270 cm^{-1} in relation to sample concentration of **MBA-BH$_3$**; m = -7.75, b = 12.26, R^2 = 0.963.

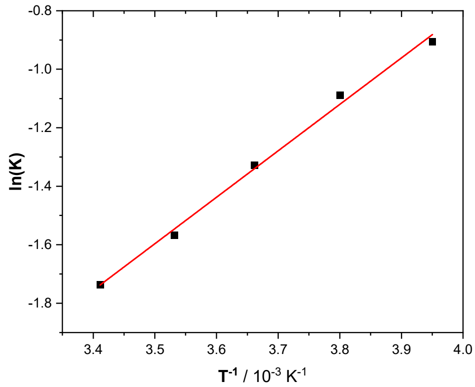

Fig. S12. MBA-BH$_3$ van't Hoff plot: Linear fit of experimental ln(K) values of **MBA-BH$_3$** against corresponding T^{-1} values. The slope was determined to be $-\Delta H \cdot R^{-1}$ = 1587.70 which corresponds to an enthalpy ΔH of -13.20 kJ·mol^{-1} or -3.16 kcal·mol^{-1}, R^2 = 0.995.

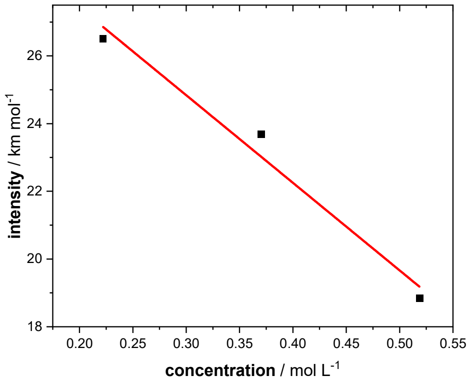

Fig. S13. BzA-BH$_3$ concentration dependence: Linear fit of integral intensity of monomer signal ~3275 cm^{-1} in relation to sample concentration of **BzA-BH$_3$**; m = -25.88, b = 32.60, R^2 = 0.977.

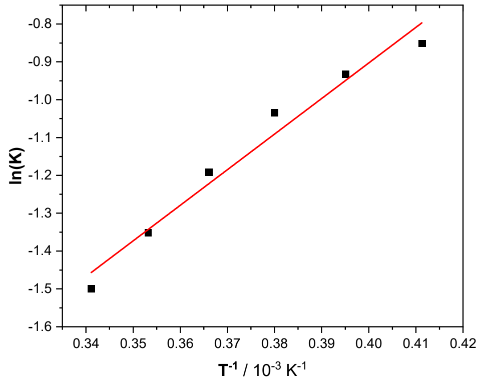

Fig. S14. BzA-BH$_3$ van't Hoff plot: Linear fit of experimental ln(K) values of **BzA-BH$_3$** against corresponding T^{-1} values. The slope was determined to be $-\Delta H \cdot R^{-1}$ = 940.51 which corresponds to an enthalpy ΔH of -7.82 kJ·mol^{-1} or -1.87 kcal·mol^{-1}, R^2 = 0.971.

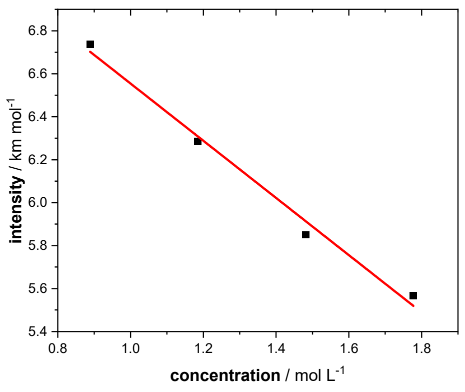

Fig. S15. Bis(MBA)-BH_3 concentration dependence: Linear fit of integral intensity of monomer signal ~3255 cm^{-1} in relation to sample concentration of **Bis(MBA)-BH_3**; m = -1.33, b = 7.89, R^2 = 0.990.

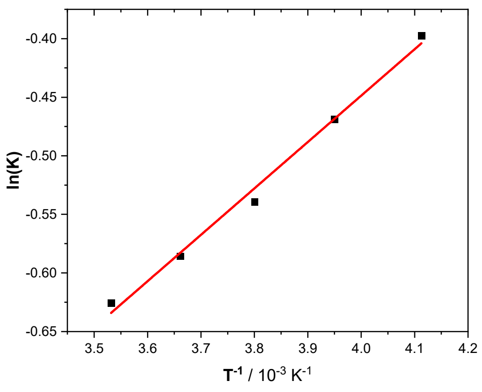

Fig. S16. Bis(MBA)-BH_3 van't Hoff plot: Linear fit of experimental ln(K) values of **Bis(MBA)-BH_3** against corresponding T^{-1} values. The slope was determined to be $-\Delta H \cdot R^{-1}$ = 396.08 which corresponds to an enthalpy ΔH of -3.29 kJ·mol^{-1} or -0.78 kcal·mol^{-1}, R^2 = 0.992.

5. Additional spectra

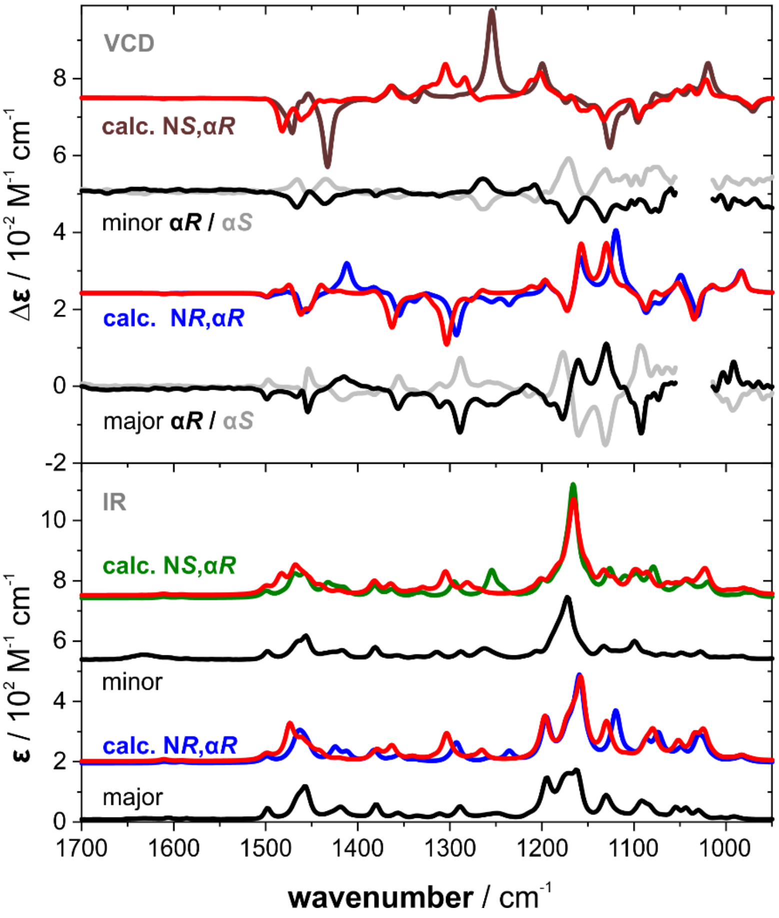

Fig. S17. Comparison of the experimental IR and VCD spectra with those calculated considering implicit (IEFPCM) solvation and by explicitly modelling a N-H···NCCD$_3$ hydrogen bond (red spcetra).

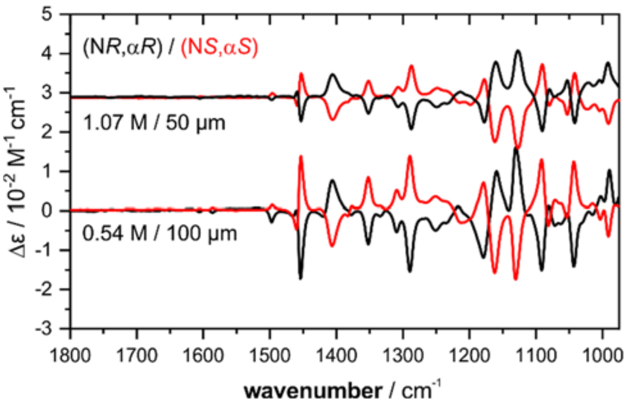

Fig. S18. VCD spectra of (N*R*,α*R*)- and (N*S*,α*S*)-**DMBA-BH₃** at different concentrations in DCM-*d*₂ at room temperature.

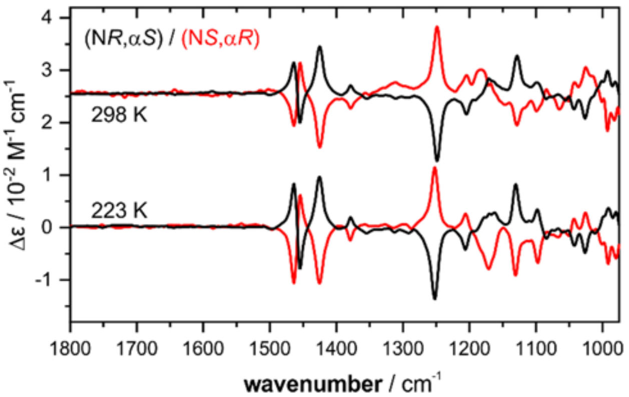

Fig. S19. VCD spectra of (N*R*,α*S*) and (N*S*,α*R*)-**DMBA-BH₃** at different temperatures in DCM-*d*₂, measured with a path length of 100 μm at a concentration of 0.3 M.

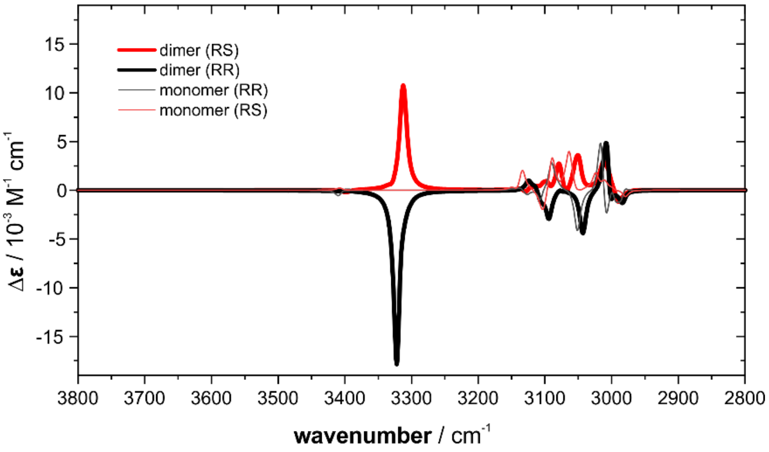

Fig. S20. VCD spectra of (N*R*,α*R*)- and (N*S*,α*S*)-**DMBA-BH₃** and their dimeric species in the N-H stretching region.

6. NCI plots

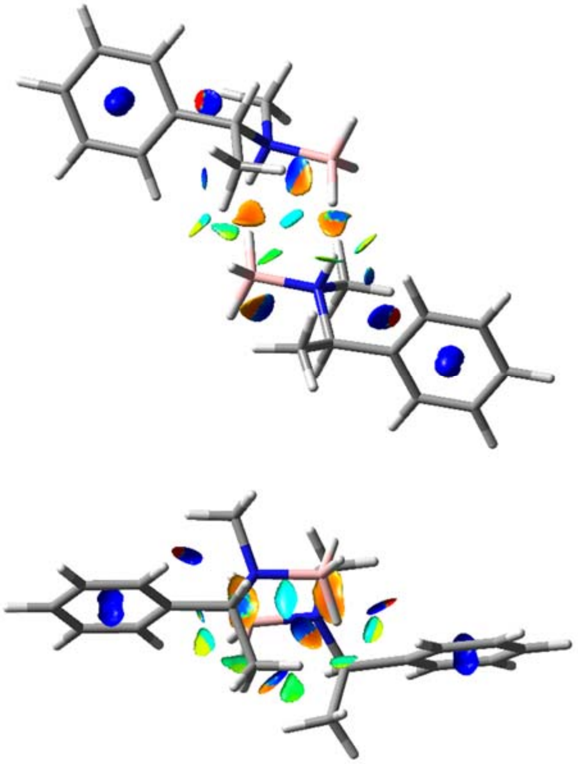

Fig. S21 NCI plots of (N*R*,α*R*)-**DMBA-BH₃** dimer

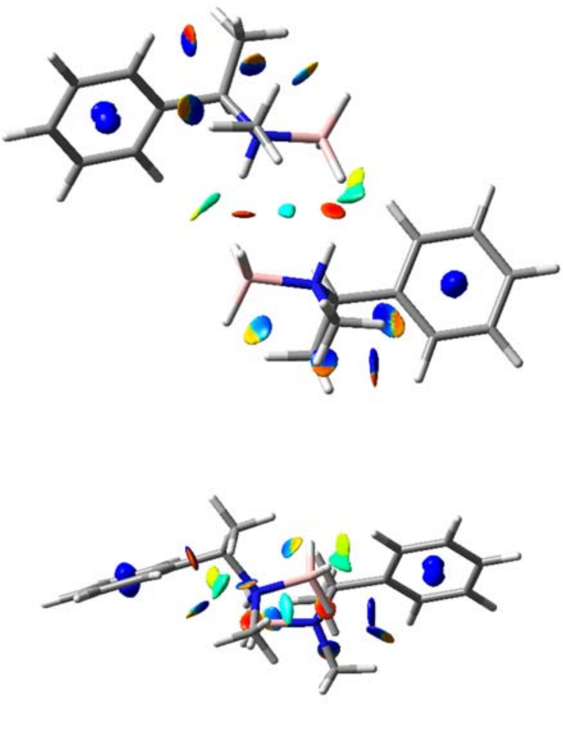

Fig. S22. NCI plots of (N*S*,α*R*)-**DMBA-BH₃** dimer

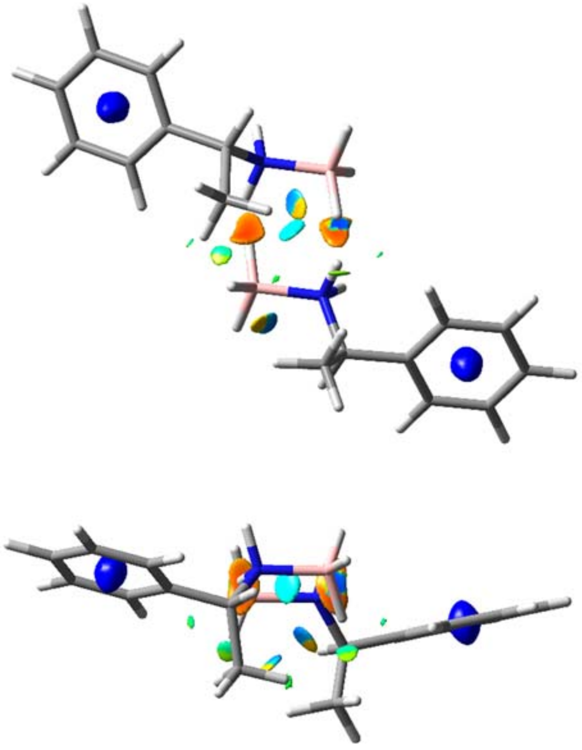

Fig. S23. NCI plots of (R)-**MBA-BH₃** dimer

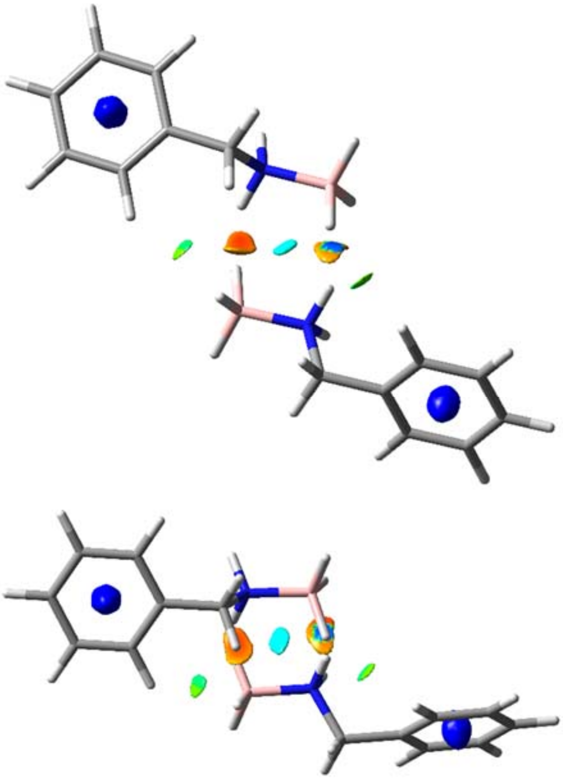

Fig. S24. NCI plots of **BzA-BH₃** dimer

7. Computational data

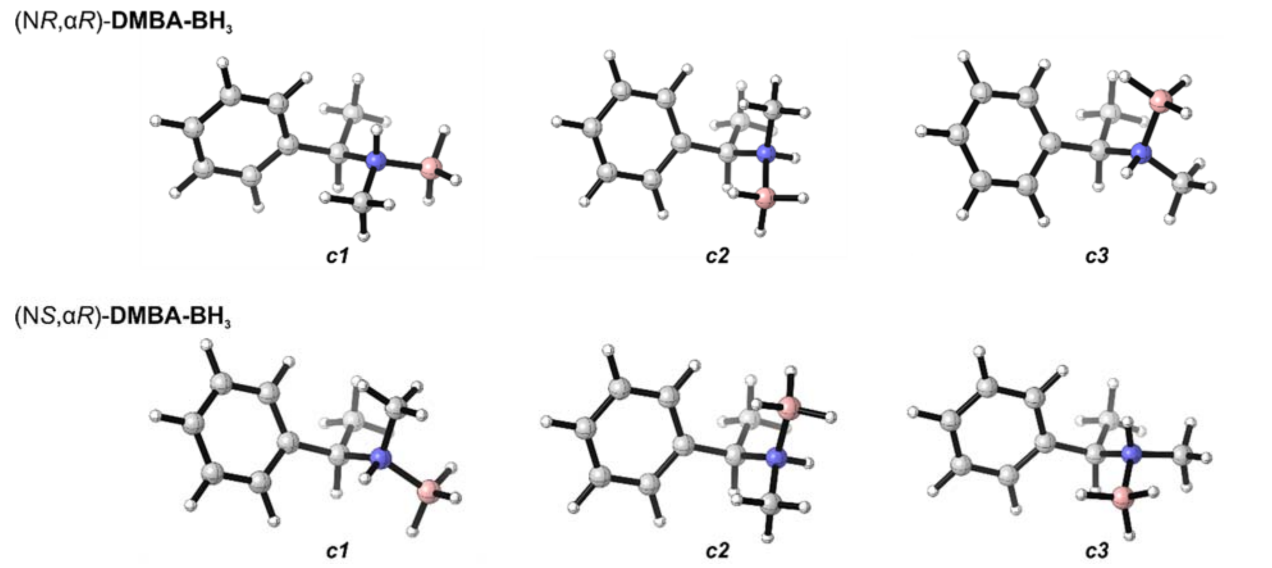

Table S1. Conformers of (N*R*,α*R*)-**DMBA-BH3** and their corresponding relative zero-point corrected energies (ΔE_{ZPC}), enthalpies (ΔH) and Gibbs free energies (ΔG_{298K}) given in kcal/mol. The Boltzmann weights χ are given in percentage. The angles α = B-N-C^{α}-C_{Ar} and β = N-C^{α}-C_{Ar}-C_{Ar} are given in degree. Calculations were carried out at B3LYP / 6-31+G(2d,p) / IEFPCM(ACN) for VCD analysis.

conf.	α	β	ΔE_{ZPC}	ΔH	ΔG_{298K}	$\chi(\Delta E_{ZPC})$	$\chi(\Delta H)$	$\chi(\Delta G_{298K})$
c1	-177.6	-119.4	0.00[a)]	0.00[b)]	0.00[c)]	80.4	80.0	83.0
c2	70.2	-98.7	1.23	1.21	1.41	10.1	10.4	7.6
c3	-78.3	-81.0	1.27	1.25	1.29	9.5	9.7	9.3

[a)] referenced to E_{ZPC}(c1) = -432.014070 hartree.
[b)] referenced to H(c1) = -432.001656 hartree.
[c)] referenced to G_{298K}(c1) = -432.051361 hartree.

Table S2. Conformers of (N*S*,α*R*)-**DMBA-BH3** and their corresponding relative zero-point corrected energies (ΔE_{ZPC}), enthalpies (ΔH) and Gibbs free energies (ΔG_{298K}) given in kcal/mol. The Boltzmann weights χ are given in percentage. The angles α = B-N-C^{α}-C_{Ar} and β = N-C^{α}-C_{Ar}-C_{Ar} are given in degree. Calculations were carried out at B3LYP / 6-31+G(2d,p) / IEFPCM(ACN) for VCD analysis.

conf.	α	β	ΔE_{ZPC}	ΔH	ΔG_{298K}	$\chi(\Delta E_{ZPC})$	$\chi(\Delta H)$	$\chi(\Delta G_{298K})$
c1	157.2	97.5	0.00[a)]	0.00[b)]	0.00[c)]	73.5	72.8	76.1
c2	-62.2	77.5	2.24	2.22	2.41	1.7	1.7	1.3
c3	66.0	62.6	0.64	0.62	0.72	24.8	25.5	22.6

[a)] referenced to E_{ZPC}(c1) = -432.013587 hartree.
[b)] referenced to H(c1) = -432.001195 hartree.
[c)] referenced to G_{298K}(c1) = -432.050813 hartree.

Table S3. Conformers of monomeric (N*R*,α*R*)-**DMBA-BH3** and their corresponding relative zero-point corrected energies (ΔE_{ZPC}), enthalpies (ΔH) and Gibbs free energies (ΔG_{298K}) given in kcal/mol. The Boltzmann weights χ are given in percentage. The angles α = B-N-C^{α}-C_{Ar} and β = N-C^{α}-C_{Ar}-C_{Ar} are given in degree. Calculations were carried out at B3LYP / 6-311++G(2d,p) / IEFPCM(DCM) for dimer analysis.

conf.	α	β	ΔE_{ZPC}	ΔH	ΔG_{298K}	χ(ΔE_{ZPC})	χ(ΔH)	χ(ΔG_{298K})
c1	-178.5	-119.3	0.00[a)]	0.00[b)]	0.00[c)]	84.8	85.0	82.1
c2	72.1	-96.4	1.46	1.45	1.33	7.3	7.3	8.7
c3	-79.5	--83.4	1.40	1.42	1.30	7.9	7.7	9.2

a) referenced to E_{ZPC}(c1) = -432.099493 hartree.
b) referenced to H(c1) = -432.087058 hartree.
c) referenced to G_{298K}(c1) = -432.136748 hartree.

Table S4. Conformers of dimeric (N*R*,α*R*)-**DMBA-BH3** and their corresponding relative zero-point corrected energies (ΔE_{ZPC}), enthalpies (ΔH) and Gibbs free energies (ΔG_{298K}) given in kcal/mol. The Boltzmann weights χ are given in percentage. Calculations were carried out at B3LYP / 6-311++G(2d,p) / IEFPCM(DCM) for dimer analysis.

conf.	ΔE_{ZPC}	ΔH	ΔG_{298K}	χ(ΔE_{ZPC})	χ(ΔH)	χ(ΔG_{298K})
HT(c1-c1)	0.00[a)]	0.00[b)]	0.00[c)]	71.4	73.9	37.9
HT(c1-c3)	1.26	1.26	0.88	8.5	8.9	8.6
HT(c1-c2)	2.04	2.03	2.04	2.3	2.4	1.2
HT(c3-c3)	2.47	2.42	2.16	1.1	1.2	1.0
HT(c2-c3)	3.34	3.30	3.23	0.3	0.3	0.2
HT(c2-c2)	4.52	4.57	4.33	0.0	0.0	0.0
open(c1-c1)-a	1.68	1.82	0.93	4.2	3.4	7.9
open(c1-c1)-b	1.62	1.80	0.45	4.6	3.5	17.8
open(c1-c1)-c	1.72	1.84	1.06	3.9	3.3	6.4
open(c1-c1)-d	2.55	2.71	1.45	1.0	0.8	3.3
open(c2-c1)-a	3.01	3.08	2.36	0.4	0.4	0.7
open(c2-c1)-b	2.98	3.05	2.25	0.5	0.4	0.9
open(c3-c1)-a	2.92	3.10	1.81	0.5	0.4	1.8
open(c2-c1)-c	3.17	3.29	2.28	0.3	0.3	0.8
open(c3-c1)-b	3.45	3.70	0.97	0.2	0.1	7.4
open(c3-c1)-c	3.63	3.83	2.09	0.2	0.1	1.1
open(c3-c1)-d	4.02	4.18	2.71	0.1	0.1	0.4
open(c3-c1)-e	4.02	4.18	2.71	0.1	0.1	0.4
open(c2-c3)-a	4.40	4.43	3.87	0.0	0.0	0.1
open(c2-c2)-a	4.28	4.38	3.15	0.1	0.1	0.2
open(c2-c2)-b	4.33	4.40	3.59	0.1	0.0	0.1
open(c2-c3)-b	4.47	4.53	4.12	0.0	0.0	0.0
open(c2-c3)-c	4.26	4.43	2.91	0.1	0.0	0.3
open(c2-c2)-c	4.55	4.65	4.01	0.0	0.0	0.0
open(c2-c2)-d	4.46	4.59	3.37	0.0	0.0	0.1
open(c2-c3)-d	4.58	4.74	3.33	0.0	0.0	0.1
open(c3-c3)-a	4.71	4.92	3.13	0.0	0.0	0.2
open(c3-c2)-a	4.72	4.90	3.63	0.0	0.0	0.1
open(c3-c2)-b	4.98	5.08	4.03	0.0	0.0	0.0
open(c3-c2)-c	4.87	5.04	3.45	0.0	0.0	0.1
open(c3-c2)-d	5.05	5.15	4.61	0.0	0.0	0.0
open(c3-c3)-b	5.04	5.24	3.46	0.0	0.0	0.1
open(c3-c3)-c	5.09	5.26	4.19	0.0	0.0	0.0
open(c3-c3)-d	5.06	5.24	3.65	0.0	0.0	0.1
open(c2-c2)-e	5.18	5.32	4.16	0.0	0.0	0.0
open(c3-c2)-e	5.35	5.54	4.30	0.0	0.0	0.0
open(c3-c3)-e	5.59	5.87	3.24	0.0	0.0	0.2
open(c3-c2)-f	5.49	5.80	2.61	0.0	0.0	0.5
open(c3-c2)-g	5.61	5.88	3.22	0.0	0.0	0.2

a) referenced to E_{ZPC}(HT(c1-c1)) = -864.203745 hartree.
b) referenced to H(HT(c1-c1)) = -864.177812 hartree.
c) referenced to G_{298K}(HT(c1-c1)) = -864.261851 hartree.

Table S5. Conformers of monomeric (N*S*,α*R*)-**DMBA-BH₃** and their corresponding relative zero-point corrected energies (ΔE_{ZPC}), enthalpies (ΔH) and Gibbs free energies (ΔG_{298K}) given in kcal/mol. The Boltzmann weights χ are given in percentage. The angles α = B-N-C^{α}-C_{Ar} and β = N-C^{α}-C_{Ar}-C_{Ar} are given in degree. Calculations were carried out at B3LYP / 6-311++G(2d,p) / IEFPCM(DCM).

conf.	α	β	ΔE_{ZPC}	ΔH	ΔG_{298K}	$\chi(\Delta E_{ZPC})$	χ(ΔH)	$\chi(\Delta G_{298K})$
c1	158.0	97.4	0.00[a)]	0.00[b)]	0.00[c)]	78.8	77.5	84.6
c2	-62.1	78.3	1.30	1.25	1.56	8.8	9.3	6.0
c3	65.9	62.8	1.10	1.05	1.31	12.4	13.2	9.3

[a)] referenced to E_{ZPC}(c1) = -432.099108 hartree.
[b)] referenced to H(c1) = -432.086657 hartree.
[c)] referenced to G_{298K}(c1) = -432.136500 hartree.

Table S6. Conformers of dimeric (N*S*,α*R*)-**DMBA-BH₃** and their corresponding relative zero-point corrected energies (ΔE_{ZPC}), enthalpies (ΔH) and Gibbs free energies (ΔG_{298K}) given in kcal/mol. The Boltzmann weights χ are given in percentage. Calculations were carried out at B3LYP / 6-311++G(2d,p) / IEFPCM(DCM) for dimer analysis.

conf.	ΔE_{ZPC}	ΔH	ΔG_{298K}	$\chi(\Delta E_{ZPC})$	χ(ΔH)	$\chi(\Delta G_{298K})$
HT(c1-c1)	0.00[a)]	0.00[b)]	0.00[c)]	47.8	52.4	15.0
HT(c1-c2)	1.05	1.04	1.26	8.1	9.1	1.8
HT(c1-c3)	1.17	1.17	1.30	6.7	7.2	1.7
HT(c2-c2)	2.10	2.02	2.56	1.4	1.7	0.2
HT(c2-c3)	2.20	2.18	2.50	1.2	1.3	0.2
HT(c3-c3)	2.58	2.62	2.97	0.6	0.6	0.1
open(c1-c1)-a	1.60	1.80	0.21	3.2	2.5	10.4
open(c1-c1)-b	1.52	1.74	0.03	3.6	2.8	14.1
open(c1-c1)-c	1.65	1.84	0.54	3.0	2.3	6.1
open(c1-c1)-d	1.75	1.89	0.99	2.5	2.1	2.8
open(c1-c1)-e	1.71	1.86	0.98	2.7	2.3	2.9
open(c1-c1)-f	1.95	2.12	1.37	1.8	1.5	1.5
open(c1-c2)-a	2.16	2.33	0.82	1.3	1.0	3.8
open(c2-c1)-a	2.11	2.29	1.08	1.4	1.1	2.4
open(c1-c1)-g	2.15	2.38	0.98	1.3	0.9	2.8
open(c1-c3)-a	2.24	2.41	1.36	1.1	0.9	1.5
open(c3-c1)-a	2.18	2.39	0.93	1.2	0.9	3.1
open(c3-c1)-b	2.13	2.35	0.66	1.3	1.0	4.9
open(c3-c1)-c	2.24	2.43	1.24	1.1	0.9	1.8
open(c1-c3)-b	2.25	2.44	1.30	1.1	0.8	1.7
open(c1-c1)-h	2.20	2.45	0.66	1.2	0.8	4.9
open(c1-c3)-c	2.18	2.40	0.77	1.2	0.9	4.1
open(c3-c1)-d	2.23	2.44	0.79	1.1	0.9	3.9
open(c1-c2)-b	2.85	2.93	2.57	0.4	0.4	0.2
open(c1-c2)-c	2.76	2.90	1.27	0.5	0.4	1.8
open(c2-c2)-a	3.38	3.45	2.31	0.2	0.2	0.3
open(c3-c3)-a	3.14	3.25	2.55	0.2	0.2	0.2
open(c3-c3)-b	3.24	3.32	2.72	0.2	0.2	0.2
open(c3-c3)-c	3.17	3.31	2.30	0.2	0.2	0.3
open(c2-c2)-b	3.41	3.52	2.33	0.2	0.1	0.3
open(c3-c3)-d	3.17	3.30	2.45	0.2	0.2	0.2
open(c2-c1)-b	3.33	3.52	1.94	0.2	0.1	0.6
open(c3-c3)-e	3.09	3.28	1.77	0.3	0.2	0.8
open(c3-c3)-f	3.21	3.36	2.52	0.2	0.2	0.2
open(c3-c3)-g	3.22	3.39	2.00	0.2	0.2	0.5
open(c2-c3)-a	3.48	3.55	3.00	0.1	0.1	0.1
open(c3-c2)-a	3.45	3.53	2.88	0.1	0.1	0.1
open(c2-c3)-b	3.32	3.49	1.75	0.2	0.1	0.8
open(c3-c2)-b	3.36	3.50	2.23	0.2	0.1	0.3
open(c3-c2)-c	3.36	3.52	2.29	0.2	0.1	0.3
open(c2-c3)-c	3.47	3.57	2.84	0.1	0.1	0.1
open(c3-c2)-d	3.47	3.60	2.70	0.1	0.1	0.2
open(c3-c2)-e	3.55	3.67	2.61	0.1	0.1	0.2
open(c2-c3)-d	3.50	3.66	2.21	0.1	0.1	0.4

open(c3-c2)-f	3.64	3.78	2.70	0.1	0.1	0.2
open(c2-c3)-e	3.81	3.93	3.14	0.1	0.1	0.1

[a] referenced to E_{ZPC}(HT(c1-c1)) = -864.201973 hartree.
[b] referenced to H(HT(c1-c1)) = -864.176038 hartree.
[c] referenced to G_{298K}(HT(c1-c1)) = -864.260667 hartree.

Table S7. Conformer energies (E_{ZPC}) and enthalpies (H) of monomeric and dimeric (N*R*,α*R*)-**DMBA-BH$_3$** and their corresponding calculated stabilization through dihydrogen bonding (ΔE_{DHB} and ΔH_{DHB}) given in Hartree as well as their product with the respective Boltzmann weights ($\chi_{dimer}\cdot\Delta E_{DHB}$ and $\chi_{dimer}\cdot\Delta H_{DHB}$) in Hartree and kcal/mol. Calculations were carried out at B3LYP / 6-311++G(2d,p) / IEFPCM(DCM).

conf.	E_{ZPC}	H	ΔE_{DHB}	ΔH_{DHB}	$\chi_{dimer}\cdot\Delta E_{DHB}$	$\chi_{dimer}\cdot\Delta H_{DHB}$	$\chi_{dimer}\cdot\Delta E_{DHB}$	$\chi_{dimer}\cdot\Delta H_{DHB}$
c1	-432.335078	-432.099493						
c2	-432.332855	-432.097174						
c3	-432.332876	-432.097254						
HT(c1-c1)	-864.203745	-864.177812	-0.004759	-0.003696	-0.003398	-0.002729	-2.13	-1.71
HT(c1-c3)	-864.201735	-864.175810	-0.004988	-0.003963	-0.000423	-0.000351	-0.27	-0.22
HT(c1-c2)	-864.200500	-864.174577	-0.003833	-0.002772	-0.000088	-0.000066	-0.06	-0.04
HT(c3-c3)	-864.199813	-864.173951	-0.005305	-0.004373	-0.000059	-0.000054	-0.04	-0.03
HT(c2-c3)	-864.198429	-864.172549	-0.004001	-0.003013	-0.000010	-0.000008	-0.01	-0.01
HT(c2-c2)	-864.196544	-864.170525	-0.002196	-0.001031	-0.000001	0.000000	0.00	0.00
open(c1-c1)-a	-864.201062	-864.174918	-0.002076	-0.000802	-0.000086	-0.000028	-0.05	-0.02
open(c1-c1)-b	-864.201160	-864.174937	-0.002174	-0.000821	-0.000100	-0.000029	-0.06	-0.02
open(c1-c1)-c	-864.201000	-864.174881	-0.002014	-0.000765	-0.000078	-0.000025	-0.05	-0.02
open(c1-c1)-d	-864.199675	-864.173498	-0.000689	0.000618	-0.000007	0.000005	0.00	0.00
open(c2-c1)-a	-864.198943	-864.172905	-0.002276	-0.001100	-0.000010	-0.000004	-0.01	0.00
open(c2-c1)-b	-864.198995	-864.172958	-0.002328	-0.001153	-0.000011	-0.000005	-0.01	0.00
open(c3-c1)-a	-864.199087	-864.172872	-0.002340	-0.001025	-0.000012	-0.000004	-0.01	0.00
open(c2-c1)-c	-864.198699	-864.172576	-0.002032	-0.000771	-0.000007	-0.000002	0.00	0.00
open(c3-c1)-b	-864.198250	-864.171922	-0.001503	-7.5E-05	-0.000003	0.000000	0.00	0.00
open(c3-c1)-c	-864.197968	-864.171713	-0.001221	0.000134	-0.000002	0.000000	0.00	0.00
open(c3-c1)-d	-864.197343	-864.171146	-0.000596	0.000701	0.000000	0.000000	0.00	0.00
open(c3-c1)-e	-864.197343	-864.171146	-0.000596	0.000701	0.000000	0.000000	0.00	0.00
open(c2-c3)-a	-864.196735	-864.170758	-0.002307	-0.001222	-0.000001	-0.000001	0.00	0.00
open(c2-c2)-a	-864.196919	-864.170832	-0.002571	-0.001338	-0.000001	-0.000001	0.00	0.00
open(c2-c2)-b	-864.196838	-864.170795	-0.002490	-0.001301	-0.000001	-0.000001	0.00	0.00
open(c2-c3)-b	-864.196628	-864.170587	-0.002200	-0.001051	-0.000001	0.000000	0.00	0.00
open(c2-c3)-c	-864.196957	-864.170749	-0.002529	-0.001213	-0.000001	-0.000001	0.00	0.00
open(c2-c2)-c	-864.196490	-864.170407	-0.002142	-0.000913	-0.000001	0.000000	0.00	0.00
open(c2-c2)-d	-864.196643	-864.170494	-0.002295	-0.001000	-0.000001	0.000000	0.00	0.00
open(c2-c3)-d	-864.196445	-864.170264	-0.002017	-0.000728	-0.000001	0.000000	0.00	0.00
open(c3-c3)-a	-864.196244	-864.169971	-0.001736	-0.000393	0.000000	0.000000	0.00	0.00
open(c3-c2)-a	-864.196228	-864.170002	-0.001800	-0.000466	0.000000	0.000000	0.00	0.00
open(c3-c2)-b	-864.195808	-864.169716	-0.001380	-0.000180	0.000000	0.000000	0.00	0.00
open(c3-c2)-c	-864.195987	-864.169786	-0.001559	-0.000250	0.000000	0.000000	0.00	0.00
open(c3-c2)-d	-864.195691	-864.169611	-0.001263	-7.5E-05	0.000000	0.000000	0.00	0.00
open(c3-c3)-b	-864.195709	-864.169463	-0.001201	0.000115	0.000000	0.000000	0.00	0.00
open(c3-c3)-c	-864.195635	-864.169433	-0.001127	0.000145	0.000000	0.000000	0.00	0.00
open(c3-c3)-d	-864.195684	-864.169460	-0.001176	0.000118	0.000000	0.000000	0.00	0.00
open(c2-c2)-e	-864.195491	-864.169331	-0.001143	0.000163	0.000000	0.000000	0.00	0.00
open(c3-c2)-e	-864.195217	-864.168977	-0.000789	0.000559	0.000000	0.000000	0.00	0.00
open(c3-c3)-e	-864.194843	-864.168460	-0.000335	0.001118	0.000000	0.000000	0.00	0.00
open(c3-c2)-f	-864.195003	-864.168571	-0.000575	0.000965	0.000000	0.000000	0.00	0.00
open(c3-c2)-g	-864.194800	-864.168440	-0.000372	0.001096	0.000000	0.000000	0.00	0.00
				$\sum (\chi_{dimer}\cdot\Delta E_{DHB} / \Delta H_{DHB})$ =	-0.004306	-0.003305	-2.70	-2.07

Table S8. Conformer energies (E_{ZPC}) and enthalpies (H) of monomeric and dimeric (N*S*,α*R*)-**DMBA-BH₃** and their corresponding calculated stabilization through dihydrogen bonding (ΔE_{DHB} and ΔH_{DHB}) given in Hartree as well as their product with the respective Boltzmann weights ($\chi_{dimer}\cdot\Delta E_{DHB}$ and $\chi_{dimer}\cdot\Delta H_{DHB}$) in Hartree and kcal/mol. Calculations were carried out at B3LYP / 6-311++G(2d,p) / IEFPCM(DCM).

conf.	E	H	ΔE_{DHB}	ΔH_{DHB}	$\chi_{dimer}\cdot\Delta E_{DHB}$	$\chi_{dimer}\cdot\Delta H_{DHB}$	$\chi_{dimer}\cdot\Delta E_{DHB}$	$\chi_{dimer}\cdot\Delta H_{DHB}$
c1	-432.334835	-432.099108						
c2	-432.332789	-432.097037						
c3	-432.332995	-432.097361						
HT(c1-c1)	-864.201973	-864.176038	-0.003757	-0.002724	-0.001797	-0.001427	-1.13	-0.90
HT(c1-c2)	-864.200301	-864.174388	-0.004156	-0.003070	-0.000338	-0.000280	-0.21	-0.18
HT(c1-c3)	-864.200113	-864.174171	-0.003644	-0.002529	-0.000243	-0.000183	-0.15	-0.11
HT(c2-c2)	-864.198619	-864.172811	-0.004545	-0.003489	-0.000062	-0.000060	-0.04	-0.04
HT(c2-c3)	-864.198462	-864.172561	-0.004064	-0.002915	-0.000047	-0.000038	-0.03	-0.02
HT(c3-c3)	-864.197856	-864.171870	-0.003134	-0.001900	-0.000019	-0.000012	-0.01	-0.01
open(c1-c1)-a	-864.199421	-864.173172	-0.001205	0.000142	-0.000039	0.000004	-0.02	0.00
open(c1-c1)-b	-864.199543	-864.173270	-0.001327	0.000044	-0.000048	0.000001	-0.03	0.00
open(c1-c1)-c	-864.199346	-864.173100	-0.001130	0.000214	-0.000033	0.000005	-0.02	0.00
open(c1-c1)-d	-864.199183	-864.173023	-0.000967	0.000291	-0.000024	0.000006	-0.02	0.00
open(c1-c1)-e	-864.199254	-864.173071	-0.001038	0.000243	-0.000028	0.000005	-0.02	0.00
open(c1-c1)-f	-864.198860	-864.172655	-0.000644	0.000659	-0.000011	0.000010	-0.01	0.01
open(c1-c2)-a	-864.198534	-864.172329	-0.002389	-0.001011	-0.000030	-0.000010	-0.02	-0.01
open(c2-c1)-a	-864.198617	-864.172394	-0.002472	-0.001076	-0.000034	-0.000012	-0.02	-0.01
open(c1-c1)-g	-864.198548	-864.172251	-0.000332	0.001063	-0.000004	0.000010	0.00	0.01
open(c1-c3)-a	-864.198410	-864.172201	-0.001941	-0.000559	-0.000021	-0.000005	-0.01	0.00
open(c3-c1)-a	-864.198500	-864.172234	-0.002031	-0.000592	-0.000024	-0.000006	-0.02	0.00
open(c3-c1)-b	-864.198576	-864.172291	-0.002107	-0.000649	-0.000028	-0.000006	-0.02	0.00
open(c3-c1)-c	-864.198404	-864.172173	-0.001935	-0.000531	-0.000021	-0.000005	-0.01	0.00
open(c1-c3)-b	-864.198382	-864.172148	-0.001913	-0.000506	-0.000020	-0.000004	-0.01	0.00
open(c1-c1)-h	-864.198472	-864.172132	-0.000256	0.001182	-0.000003	0.000010	0.00	0.01
open(c1-c3)-c	-864.198495	-864.172210	-0.002026	-0.000568	-0.000024	-0.000005	-0.02	0.00
open(c3-c1)-d	-864.198427	-864.172152	-0.001958	-0.000510	-0.000022	-0.000004	-0.01	0.00
open(c1-c2)-b	-864.197431	-864.171374	-0.001286	-0.000056	-0.000005	0.000000	0.00	0.00
open(c1-c2)-c	-864.197567	-864.171420	-0.001422	-0.000102	-0.000006	0.000000	0.00	0.00
open(c2-c2)-a	-864.196592	-864.170543	-0.002518	-0.001221	-0.000004	-0.000002	0.00	0.00
open(c3-c3)-a	-864.196969	-864.170863	-0.002247	-0.000893	-0.000005	-0.000002	0.00	0.00
open(c3-c3)-b	-864.196813	-864.170740	-0.002091	-0.000770	-0.000004	-0.000001	0.00	0.00
open(c3-c3)-c	-864.196925	-864.170760	-0.002203	-0.000790	-0.000005	-0.000002	0.00	0.00
open(c2-c2)-b	-864.196538	-864.170429	-0.002464	-0.001107	-0.000004	-0.000002	0.00	0.00
open(c3-c3)-d	-864.196929	-864.170780	-0.002207	-0.000810	-0.000005	-0.000002	0.00	0.00
open(c2-c1)-b	-864.196661	-864.170429	-0.000516	0.000889	-0.000001	0.000001	0.00	0.00
open(c3-c3)-e	-864.197050	-864.170807	-0.002328	-0.000837	-0.000006	-0.000002	0.00	0.00
open(c3-c3)-f	-864.196863	-864.170687	-0.002141	-0.000717	-0.000005	-0.000001	0.00	0.00
open(c3-c3)-g	-864.196848	-864.170643	-0.002126	-0.000673	-0.000004	-0.000001	0.00	0.00
open(c2-c3)-a	-864.196434	-864.170382	-0.002036	-0.000736	-0.000003	-0.000001	0.00	0.00
open(c3-c2)-a	-864.196472	-864.170412	-0.002074	-0.000766	-0.000003	-0.000001	0.00	0.00
open(c2-c3)-b	-864.196677	-864.170476	-0.002279	-0.000830	-0.000004	-0.000001	0.00	0.00
open(c3-c2)-b	-864.196616	-864.170464	-0.002218	-0.000818	-0.000004	-0.000001	0.00	0.00
open(c3-c2)-c	-864.196618	-864.170433	-0.002220	-0.000787	-0.000004	-0.000001	0.00	0.00
open(c2-c3)-c	-864.196447	-864.170352	-0.002049	-0.000706	-0.000003	-0.000001	0.00	0.00
open(c3-c2)-d	-864.196445	-864.170301	-0.002047	-0.000655	-0.000003	-0.000001	0.00	0.00
open(c3-c2)-e	-864.196323	-864.170193	-0.001925	-0.000547	-0.000002	-0.000001	0.00	0.00
open(c2-c3)-d	-864.196401	-864.170213	-0.002003	-0.000567	-0.000003	-0.000001	0.00	0.00
open(c3-c2)-f	-864.196170	-864.170013	-0.001772	-0.000367	-0.000002	0.000000	0.00	0.00
open(c2-c3)-e	-864.195906	-864.169768	-0.001508	-0.000122	-0.000001	0.000000	0.00	0.00
				$\sum (\chi_{dimer}\cdot\Delta E_{DHB} / \Delta H_{DHB}) =$	-0.003006	-0.002030	-1.89	-1.27

Table S9. Conformers of monomeric (*R*)-**MBA-BH3** and its corresponding relative zero-point corrected energies (ΔE_{ZPC}), enthalpies (ΔH) and Gibbs free energies (ΔG_{298K}) given in kcal/mol. The Boltzmann weights χ are given in percentage. The angles α = B-N-C$^{\alpha}$-C$_{Ar}$ and β = N-C$^{\alpha}$-C$_{Ar}$-C$_{Ar}$ are given in degree. Calculations were carried out at B3LYP / 6-311++G(2d,p) / IEFPCM(DCM) for dimer analysis.

conf.	α	β	ΔE_{ZPC}	ΔH	ΔG_{298K}	$\chi(\Delta E_{ZPC})$	$\chi(\Delta H)$	$\chi(\Delta G_{298K})$
c1	-71.2	-89.7	2.63	2.55	2.87	0.8	0.9	0.5
c2	162.4	-113.6	0.00 [a)]	0.00 [b)]	0.00 [c)]	68.4	68.4	69.1
c3	74.4	-118.0	0.47	0.48	0.49	30.8	30.7	30.4

[a)] referenced to E_{ZPC}(c1) = -392.815121 hartree.
[b)] referenced to H(c1) = -392.851340 hartree.
[c)] referenced to G_{298K}(c1) = -392.837264 hartree.

Table S10. Conformers of dimeric (*R*)-**MBA-BH3** and their corresponding relative zero-point corrected energies (ΔE_{ZPC}), enthalpies (ΔH) and Gibbs free energies (ΔG_{298K}) given in kcal/mol. The Boltzmann weights χ are given in percentage. Calculations were carried out at B3LYP / 6-311++G(2d,p) / IEFPCM(DCM) for dimer analysis.

conf.	ΔE_{ZPC}	ΔH	ΔG_{298K}	$\chi(\Delta E_{ZPC})$	$\chi(\Delta H)$	$\chi(\Delta G_{298K})$
HT(c2-c2)-a	0.00 [a)]	0.00 [b)]	1.48	22.6	20.7	6.8
HT(c2-c2)-b	0.21	0.16	1.77	15.9	15.7	4.1
HT(c2-c3)-a	0.35	0.26	2.26	12.5	13.4	1.8
HT(c2-c2)-c	0.19	0.24	0.00 [c)]	16.3	13.7	82.4
HT(c2-c3)-b	0.64	0.49	2.86	7.7	9.0	0.7
HT(c3-c3)-a	0.74	0.56	2.55	6.4	8.0	1.1
HT(c3-c2)-a	0.64	0.57	2.33	7.7	7.9	1.6
HT(c3-c2)-b	0.93	0.84	2.89	4.7	5.0	0.6
HT(c3-c3)-b	0.95	0.84	3.10	4.5	5.0	0.4
HT(c3-c3)-c	1.72	1.68	3.61	1.2	1.2	0.2
open(c3-c2)-a	2.51	2.62	3.49	0.3	0.2	0.2

[a)] referenced to E_{ZPC}(HT(c2-c2)-a) = -785.634826 hartree.
[b)] referenced to H(HT(c2-c2)-a) = -785.611273 hartree.
[c)] referenced to G_{298K}(HT(c2-c2)-a) = -785.693845 hartree.

Table S11. Conformer energies (E_{ZPC}) and enthalpies (H) of monomeric and dimeric (*R*)-**MBA-BH3** and their corresponding calculated stabilization through dihydrogen bonding (ΔE_{DHB} and ΔH_{DHB}) given in Hartree as well as their product with the respective Boltzmann weights ($\chi_{dimer}\cdot\Delta E_{DHB}$ and $\chi_{dimer}\cdot\Delta H_{DHB}$) in Hartree and kcal/mol. Calculations were carried out at B3LYP / 6-311++G(2d,p) / IEFPCM(DCM).

conf.	E	H	ΔE_{DHB}	ΔH_{DHB}	$\chi_{dimer}\cdot\Delta E_{DHB}$	$\chi_{dimer}\cdot\Delta H_{DHB}$	$\chi_{dimer}\cdot\Delta E_{DHB}$	$\chi_{dimer}\cdot\Delta H_{DHB}$
c1	-392.810936	-392.799773						
c2	-392.815121	-392.803841						
c3	-392.814369	-392.803084						
HT(c2-c2)-a	-785.634826	-785.611273	-0.004584	-0.003591	-0.001034	-0.000744	-0.65	-0.47
HT(c2-c2)-b	-785.634496	-785.611013	-0.004254	-0.003331	-0.000677	-0.000524	-0.42	-0.33
HT(c2-c3)-a	-785.63427	-785.610865	-0.004780	-0.003940	-0.000598	-0.000530	-0.38	-0.33
HT(c2-c2)-c	-785.634522	-785.610884	-0.004280	-0.003202	-0.000700	-0.000439	-0.44	-0.28
HT(c2-c3)-b	-785.633813	-785.610485	-0.004323	-0.003560	-0.000333	-0.000320	-0.21	-0.20
HT(c3-c3)-a	-785.633643	-785.610381	-0.004905	-0.004213	-0.000316	-0.000339	-0.20	-0.21
HT(c3-c2)-a	-785.633812	-785.610364	-0.004322	-0.003439	-0.000333	-0.000272	-0.21	-0.17
HT(c3-c2)-b	-785.63335	-785.609933	-0.003860	-0.003008	-0.000182	-0.000151	-0.11	-0.09
HT(c3-c3)-b	-785.63331	-785.609934	-0.004572	-0.003766	-0.000207	-0.000189	-0.13	-0.12
HT(c3-c3)-c	-785.632078	-785.608597	-0.003340	-0.002429	-0.000041	-0.000030	-0.03	-0.02
open(c3-c2)-a	-785.630834	-785.607103	-0.001344	-0.000178	-0.000004	0.000000	0.00	0.00
				$\sum (\chi_{dimer}\cdot\Delta E_{DHB} / \Delta H_{DHB})$ =	-0.004426	-0.003536	-2.78	-2.22

Table S12. Conformer energies (E_{ZPC}) and enthalpies (H) of monomeric and dimeric (*R*)-**MBA-BH₃** and their corresponding relative zero-point corrected energies (ΔE_{ZPC}) and enthalpies (ΔH) given in Hartree and kcal/mol, respectively. Calculations were carried out at B3LYP / 6-311++G(2d,p) / SMD(DCM) and B3LYP / 6-311++G(2d,p) / IEFPCM(DCM) with GD3BJ dispersion correction.

**6-311++G(2d,p) / SMD(DCM)**					**6-311++G(2d,p) / IEFPCM(DCM) with GD3BJ**				
**conf.**	**E_{ZPC}**	**H**	**ΔE_{ZPC}**	**ΔH**	**conf.**	**E_{ZPC}**	**H**	**ΔE_{ZPC}**	**ΔH**
c2	-392.828253	-392.817025	0.00	0.00	c2	-392.85717	-392.845889	0.00	0.00
c3	-392.827497	-392.816334	0.45	0.79	c3	-392.85707	-392.845731	0.06	0.10
c1	-392.823939	-392.8129	0.53	0.85	c1	-392.85394	-392.84281	2.03	1.93
HT(c2-c2)-a	-785.659272	-785.636292	0.00	0.00	HT(c3-c2)-a	-785.732403	-785.709694	0.00	0.00
HT(c2-c2)-b	-785.658558	-785.635034	0.45	0.79	HT(c3-c2)-b	-785.730059	-785.707253	1.47	1.53
HT(c2-c3)-a	-785.658435	-785.634944	0.53	0.85	HT(c3-c3)-b	-785.729986	-785.707193	1.52	1.57
HT(c3-c2)-a	-785.658266	-785.634818	0.63	0.92	HT(c3-c3)-c	-785.728659	-785.70582	2.35	2.43
HT(c2-c3)-b	-785.658094	-785.634649	0.74	1.03	HT(c2-c2)-a	-785.728135	-785.705052	2.68	2.91
HT(c2-c2)-c	-785.658507	-785.634722	0.48	0.99	HT(c2-c3)-a	-785.727334	-785.704317	3.18	3.37
HT(c3-c3)-a	-785.657668	-785.634459	1.01	1.15	HT(c2-c2)-b	-785.726996	-785.703953	3.39	3.60
HT(c3-c3)-b	-785.657655	-785.634314	1.01	1.24	HT(c2-c2)-c	-785.72657	-785.703564	3.66	3.85
HT(c3-c2)-b	-785.658052	-785.634553	0.77	1.09	HT(c3-c3)-a	-785.726379	-785.703599	3.78	3.82
HT(c3-c3)-c	-785.657771	-785.634094	0.94	1.38	HT(c2-c3)-b	-785.72666	-785.703629	3.60	3.81
open(c3-c2)-a	-785.657248	-785.633476	1.27	1.77	open(c3-c2)-a	-785.720326	-785.69697	7.58	7.98

Table S13. Stabilization of the dimeric conformers of (*R*)-**MBA-BH₃** through dihydrogen bonding (ΔE_{DHB} and ΔH_{DHB}) given in Hartree as well as their product with the respective Boltzmann weights ($\chi_{dimer}\cdot\Delta E_{DHB}$ and $\chi_{dimer}\cdot\Delta H_{DHB}$) in kcal/mol. Calculations were carried out at B3LYP / 6-311++G(2d,p) / SMD(DCM) and B3LYP / 6-311++G(2d,p) / IEFPCM(DCM) with GD3BJ dispersion correction.

**6-311++G(2d,p) / SMD(DCM)**					**6-311++G(2d,p) / IEFPCM(DCM) with GD3BJ**				
**conf.**	**ΔE_{DHB}**	**ΔH_{DHB}**	**$\chi_{dimer}\cdot\Delta E_{DHB}$**	**$\chi_{dimer}\cdot\Delta H_{DHB}$**	**conf.**	**ΔE_{DHB}**	**ΔH_{DHB}**	**$\chi_{dimer}\cdot\Delta E_{DHB}$**	**$\chi_{dimer}\cdot\Delta H_{DHB}$**
HT(c2-c2)-a	-0.002766	-0.002242	-0.44	-0.53	HT(c3-c2)-a	-0.018163	-0.018074	-9.46	-9.61
HT(c2-c2)-b	-0.002052	-0.000984	-0.15	-0.06	HT(c3-c2)-b	-0.015819	-0.015633	-0.69	-0.63
HT(c2-c3)-a	-0.002685	-0.001585	-0.18	-0.09	HT(c3-c3)-b	-0.015846	-0.015731	-0.64	-0.59
HT(c2-c3)-a	-0.002516	-0.001459	-0.14	-0.07	HT(c3-c3)-c	-0.014519	-0.014358	-0.14	-0.13
HT(c3-c2)-a	-0.002344	-0.00129	-0.11	-0.05	HT(c2-c2)-a	-0.013795	-0.013274	-0.08	-0.05
HT(c2-c2)-c	-0.002001	-0.000672	-0.14	-0.03	HT(c2-c3)-a	-0.013094	-0.012697	-0.03	-0.02
HT(c3-c3)-a	-0.002674	-0.001791	-0.08	-0.06	HT(c2-c2)-b	-0.012656	-0.012175	-0.02	-0.01
HT(c3-c3)-b	-0.002661	-0.001646	-0.08	-0.05	HT(c2-c2)-c	-0.01223	-0.011786	-0.01	-0.01
HT(c3-c2)-b	-0.002302	-0.001194	-0.10	-0.04	HT(c3-c3)-a	-0.012239	-0.012137	-0.01	-0.01
HT(c3-c3)-c	-0.002777	-0.001426	-0.09	-0.03	HT(c2-c3)-b	-0.01242	-0.012009	-0.01	-0.01
open(c3-c2)-a	-0.001498	-0.000117	-0.03	0.00	open(c2-c3)-a	-0.006086	-0.00535	0.00	0.00
	**$\sum$ ($\chi_{dimer}\cdot\Delta E_{DHB}$ / ΔH_{DHB}) =**		-1.54	-1.03		**$\sum$ ($\chi_{dimer}\cdot\Delta E_{DHB}$ / ΔH_{DHB}) =**		-11.10	-11.07

Table S14. Conformers of monomeric **BzA-BH₃** and its corresponding relative zero-point corrected energies (ΔE_{ZPC}), enthalpies (ΔH) and Gibbs free energies (ΔG_{298K}) given in kcal/mol. The Boltzmann weights χ are given in percentage. The angles α = B-N-C^{α}-C_{Ar} and β = N-C^{α}-C_{Ar}-C_{Ar} are given in degree. Calculations were carried out at B3LYP / 6-311++G(2d,p) / IEFPCM(DCM) for dimer analysis.

**conf.**	**α-1**	**β-1**	**ΔE_{ZPC}**	**ΔH**	**ΔG_{298K}**	**$\chi(\Delta E_{ZPC})$**	**$\chi(\Delta H)$**	**$\chi(\Delta G_{298K})$**
c1	-179.6	-89.0	0.00 [a)]	0.00 [b)]	0.00 [c)]	94.8	94.4	96.1
c2	75.4	-99.9	2.14	2.09	2.35	2.5	2.8	1.8
c3	-77.9	-80.5	2.12	2.07	2.27	2.6	2.9	2.1

[a)] referenced to E_{ZPC}(c1) = -353.518780 hartree.
[b)] referenced to H(c1) = -353.508860 hartree.
[c)] referenced to G_{298K} (c1) = -353.553394 hartree.

Table S15. Conformers of dimeric of **BzA-BH$_3$** and its corresponding relative zero-point corrected energies (ΔE_{ZPC}), enthalpies (ΔH) and Gibbs free energies (ΔG_{298K}) given in kcal/mol. The Boltzmann weights χ are given in percentage. Calculations were carried out at B3LYP / 6-311++G(2d,p) / IEFPCM(DCM) for dimer analysis.

**conf.**	**ΔE_{ZPC}**	**ΔH**	**ΔG_{298K}**	**$\chi(\Delta E_{ZPC})$**	**$\chi(\Delta H)$**	**$\chi(\Delta G_{298K})$**
HT(c1-c1)-a	0.00[a)]	0.00[b)]	0.00[c)]	46.5	44.3	60.2
HT(c1-c1)-b	0.09	0.04	0.51	39.7	41.4	25.2
HT(c1-c2)-a	1.78	1.75	1.89	2.3	2.3	2.5
HT(c1-c2)-b	1.89	1.81	2.47	1.9	2.1	0.9
HT(c1-c3)-a	1.90	1.80	2.23	1.9	2.1	1.4
HT(c1-c3)-b	1.91	1.81	2.54	1.9	2.1	0.8
open(c1-c1)-a	2.21	2.25	2.90	1.1	1.0	0.4
HT(c1-c3)-c	2.25	2.22	2.22	1.0	1.0	1.4
HT(c1-c2)-c	2.29	2.25	2.38	1.0	1.0	1.1
HT(c1-c3)-d	2.34	2.27	2.22	0.9	1.0	1.4
HT(c1-c2)-d	2.37	2.30	2.44	0.9	0.9	1.0
open(c2-c1)-a	3.20	3.34	2.29	0.2	0.2	1.3
HT(c2-c3)-a	3.56	3.51	3.62	0.1	0.1	0.1
HT(c3-c3)-a	3.61	3.52	4.16	0.1	0.1	0.1
open(c3-c1)-a	3.68	3.88	2.29	0.1	0.1	1.2
HT(c2-c3)-b	4.01	3.94	4.20	0.1	0.1	0.1
HT(c3-c3)-b	4.01	3.97	3.94	0.1	0.1	0.1
open(c1-c2)-a	4.03	4.08	4.41	0.1	0.0	0.0
open(c3-c1)-b	4.03	4.08	4.41	0.1	0.0	0.0
HT(c2-c2)-a	4.04	3.98	3.77	0.1	0.1	0.1
HT(c2-c3)-c	4.07	3.97	4.34	0.0	0.1	0.0
open(c3-c1)-c	4.10	4.23	3.60	0.0	0.0	0.1
HT(c2-c3)-d	4.47	4.41	3.90	0.0	0.0	0.1
HT(c2-c2)-b	4.94	4.95	4.95	0.0	0.0	0.0
HT(c3-c3)-c	4.95	4.96	5.11	0.0	0.0	0.0
HT(c3-c3)-d	5.12	5.04	5.59	0.0	0.0	0.0
open(c2-c3)-a	5.15	5.28	4.64	0.0	0.0	0.0
open(c2-c3)-b	5.18	5.29	4.69	0.0	0.0	0.0
open(c2-c2)-a	5.20	5.37	3.59	0.0	0.0	0.1
open(c2-c3)-c	5.22	5.31	4.99	0.0	0.0	0.0
open(c2-c2)-b	5.22	5.37	4.26	0.0	0.0	0.0
open(c2-c2)-c	5.60	5.76	4.66	0.0	0.0	0.0
open(c3-c2)-a	5.81	5.95	5.34	0.0	0.0	0.0
open(c3-c2)-b	5.85	5.97	5.62	0.0	0.0	0.0

[a)] referenced to E_{ZPC}(HT(c1-c1)-a) = -707.042296 hartree.
[b)] referenced to H(HT(c1-c1)-a) = -707.021600 hartree.
[c)] referenced to G_{298K}(HT(c1-c1)-a) = -707.096241 hartree.

Table S16. Conformer energies (E_{ZPC}) and enthalpies (H) of monomeric and dimeric **BzA-BH₃** and their corresponding calculated stabilization through dihydrogen bonding (ΔE_{DHB} and ΔH_{DHB}) given in Hartree as well as their product with the respective Boltzmann weights ($\chi_{dimer} \cdot \Delta E_{DHB}$ and $\chi_{dimer} \cdot \Delta H_{DHB}$) in Hartree and kcal/mol. Calculations were carried out at B3LYP / 6-311++G(2d,p) / IEFPCM(DCM).

conf.	E	H	ΔE_{DHB}	ΔH_{DHB}	$\chi_{dimer} \cdot \Delta E_{DHB}$	$\chi_{dimer} \cdot \Delta H_{DHB}$	$\chi_{dimer} \cdot \Delta E_{DHB}$	$\chi_{dimer} \cdot \Delta H_{DHB}$
c1	-432.334835	-432.099108						
c2	-432.332789	-432.097037						
c3	-432.332995	-432.097361						
HT(c1-c1)-a	-707.042296	-707.021600	-0.004736	-0.003880	-0.002203	-0.001717	-1.38	-1.08
HT(c1-c1)-b	-707.042146	-707.021536	-0.004586	-0.003816	-0.001820	-0.001578	-1.14	-0.99
HT(c1-c2)-a	-707.039454	-707.018818	-0.005312	-0.004424	-0.000122	-0.000103	-0.08	-0.06
HT(c1-c2)-b	-707.039279	-707.018722	-0.005137	-0.004328	-0.000098	-0.000091	-0.06	-0.06
HT(c1-c3)-a	-707.039272	-707.018728	-0.005130	-0.004334	-0.000097	-0.000091	-0.06	-0.06
HT(c1-c3)-b	-707.039256	-707.018713	-0.005114	-0.004319	-0.000095	-0.000090	-0.06	-0.06
open(c1-c1)-a	-707.038778	-707.018007	-0.004598	-0.003585	-0.000051	-0.000035	-0.03	-0.02
HT(c1-c3)-c	-707.038710	-707.018055	-0.004530	-0.003633	-0.000047	-0.000038	-0.03	-0.02
HT(c1-c2)-c	-707.038644	-707.018019	-0.004464	-0.003597	-0.000043	-0.000036	-0.03	-0.02
HT(c1-c3)-d	-707.038573	-707.017978	-0.004393	-0.003556	-0.000040	-0.000034	-0.02	-0.02
HT(c1-c2)-d	-707.038520	-707.017935	-0.007796	-0.006867	-0.000066	-0.000063	-0.04	-0.04
open(c2-c1)-a	-707.037196	-707.016273	-0.006472	-0.005205	-0.000014	-0.000008	-0.01	-0.01
HT(c2-c3)-a	-707.036618	-707.016006	-0.005856	-0.004910	-0.000007	-0.000006	0.00	0.00
HT(c3-c3)-a	-707.036538	-707.015994	-0.005776	-0.004898	-0.000006	-0.000006	0.00	0.00
open(c3-c1)-a	-707.036432	-707.015423	-0.005670	-0.004327	-0.000005	-0.000003	0.00	0.00
HT(c2-c3)-b	-707.035901	-707.015319	-0.005139	-0.004223	-0.000003	-0.000002	0.00	0.00
HT(c3-c3)-b	-707.035901	-707.015275	-0.005101	-0.004151	-0.000003	-0.000002	0.00	0.00
open(c1-c2)-a	-707.035868	-707.015100	-0.005068	-0.003976	-0.000003	-0.000002	0.00	0.00
open(c3-c1)-b	-707.035868	-707.015100	-0.005068	-0.003976	-0.000003	-0.000002	0.00	0.00
HT(c2-c2)-a	-707.035854	-707.015261	-0.005054	-0.004137	-0.000003	-0.000002	0.00	0.00
HT(c2-c3)-c	-707.035806	-707.015268	0.001754	0.002452	0.000001	0.000001	0.00	0.00
open(c3-c1)-c	-707.035770	-707.014863	-0.001628	-0.000469	-0.000001	0.000000	0.00	0.00
HT(c2-c3)-d	-707.035170	-707.014575	-0.001028	-0.000181	0.000000	0.000000	0.00	0.00
HT(c2-c2)-b	-707.034422	-707.013710	-0.003698	-0.002642	0.000000	0.000000	0.00	0.00
HT(c3-c3)-c	-707.034401	-707.013696	-0.003677	-0.002628	0.000000	0.000000	0.00	0.00
HT(c3-c3)-d	-707.034142	-707.013572	-0.003418	-0.002504	0.000000	0.000000	0.00	0.00
open(c2-c3)-a	-707.034089	-707.013192	-0.003327	-0.002096	0.000000	0.000000	0.00	0.00
open(c2-c3)-b	-707.034043	-707.013170	-0.003281	-0.002074	0.000000	0.000000	0.00	0.00
open(c2-c2)-a	-707.034006	-707.013050	-0.003244	-0.001954	0.000000	0.000000	0.00	0.00
open(c2-c3)-c	-707.033981	-707.013139	0.000199	0.001283	0.000000	0.000000	0.00	0.00
open(c2-c2)-b	-707.033972	-707.013042	0.000208	0.001380	0.000000	0.000000	0.00	0.00
open(c2-c2)-c	-707.033368	-707.012425	0.000812	0.001997	0.000000	0.000000	0.00	0.00
open(c3-c2)-a	-707.033031	-707.012121	-0.002269	-0.001025	0.000000	0.000000	0.00	0.00
open(c3-c2)-b	-707.032977	-707.012090	-0.002215	-0.000994	0.000000	0.000000	0.00	0.00
				$\sum (\chi_{dimer} \cdot \Delta E_{DHB} / \Delta H_{DHB}) =$	-0.004729	-0.003908	-2.97	-2.45

Table S17. Conformer energies (E_{ZPC}) and enthalpies (H) of monomeric and dimeric **BzA-BH3** and their corresponding relative zero-point corrected energies (ΔE_{ZPC}) and enthalpies (ΔH) given in Hartree and kcal/mol, respectively. Calculations were carried out at B3LYP / 6-311++G(2d,p) / SMD(DCM) and B3LYP / 6-311++G(2d,p) / IEFPCM(DCM) with GD3BJ dispersion correction.

**6-311++G(2d,p) / SMD(DCM)**					**6-311++G(2d,p) / IEFPCM(DCM) with GD3BJ**				
**conf.**	**E_{ZPC}**	**H**	**ΔE_{ZPC}**	**ΔH**	**conf.**	**E_{ZPC}**	**H**	**ΔE_{ZPC}**	**ΔH**
c1	-353.531597	-353.521695	0.00	0.00	c1	-353.553561	-353.543665	0.00	0.00
c2	-353.528049	-353.518265	2.23	2.15	c2	-353.551134	-353.541359	1.52	1.45
c3	-353.527959	-353.518197	2.28	2.20	c3	-353.55119	-353.541406	1.49	1.42
HT(c1-c1)-a	-707.065213	-707.044452	0.00	0.02	HT(c1-c3)-c	-707.123254	-707.103313	0.00	0.00
HT(c1-c1)-b	-707.065166	-707.044477	0.03	0.00	HT(c1-c2)-d	-707.123127	-707.103223	0.08	0.06
open(c1-c1)-a	-707.063187	-707.042138	1.27	1.47	open(c1-c1)-a	-707.120684	-707.100361	1.61	1.85
HT(c1-c2)-b	-707.062458	-707.041713	1.73	1.73	open(c3-c1)-c	-707.120653	-707.100556	1.63	1.73
HT(c1-c2)-a	-707.062426	-707.041728	1.75	1.73	HT(c1-c3)-d	-707.120539	-707.100467	1.70	1.79
HT(c1-c3)-a	-707.062344	-707.041666	1.80	1.76	HT(c1-c2)-c	-707.120392	-707.100424	1.80	1.81
HT(c1-c2)-d	-707.062103	-707.041337	1.95	1.97	HT(c1-c1)-a	-707.119685	-707.099425	2.24	2.44
HT(c1-c3)-b	-707.062101	-707.041506	1.95	1.86	HT(c1-c1)-b	-707.119661	-707.099425	2.25	2.44
HT(c1-c3)-d	-707.062091	-707.04138	1.96	1.94	HT(c3-c3)-b	-707.118565	-707.098529	2.94	3.00
HT(c1-c3)-c	-707.062063	-707.041348	1.98	1.96	HT(c3-c3)-c	-707.118514	-707.098508	2.97	3.02
HT(c1-c2)-c	-707.061864	-707.041288	2.10	2.00	HT(c2-c2)-a	-707.118357	-707.098402	3.07	3.08
open(c2-c1)-a	-707.061275	-707.040341	2.47	2.60	HT(c2-c3)-d	-707.118152	-707.09816	3.20	3.23
open(c3-c1)-c	-707.060746	-707.039688	2.80	3.01	HT(c2-c3)-c	-707.118118	-707.098139	3.22	3.25
open(c3-c1)-a	-707.060741	-707.039835	2.81	2.91	HT(c1-c2)-a	-707.117245	-707.097057	3.77	3.93
open(c1-c2)-a	-707.059574	-707.038815	3.54	3.55	HT(c1-c3)-a	-707.117212	-707.097037	3.79	3.94
open(c3-c1)-b	-707.059574	-707.038815	3.54	3.55	HT(c1-c3)-b	-707.117165	-707.096936	3.82	4.00
HT(c3-c3)-a	-707.059447	-707.038795	3.62	3.57	HT(c1-c2)-b	-707.117126	-707.096914	3.85	4.02
HT(c2-c3)-a	-707.059371	-707.039781	3.67	2.95	HT(c3-c3)-d	-707.116931	-707.09681	3.97	4.08
HT(c3-c3)-b	-707.059334	-707.03866	3.69	3.65	HT(c2-c2)-b	-707.116792	-707.096728	4.05	4.13
HT(c3-c3)-c	-707.059085	-707.038484	3.85	3.76	open(c3-c1)-c	-707.116145	-707.095715	4.46	4.77
HT(c2-c2)-a	-707.059016	-707.038453	3.89	3.78	open(c1-c2)-a	-707.116145	-707.095715	4.46	4.77
HT(c2-c3)-b	-707.059015	-707.038499	3.89	3.75	open(c3-c2)-b	-707.114904	-707.094663	5.24	5.43
HT(c3-c3)-d	-707.05901	-707.038528	3.89	3.73	open(c3-c2)-a	-707.114897	-707.09465	5.24	5.44
HT(c2-c3)-c	-707.058877	-707.038431	3.98	3.79	HT(c2-c3)-a	-707.114795	-707.094528	5.31	5.51
HT(c2-c3)-d	-707.05884	-707.038209	4.00	3.93	open(c2-c3)-c	-707.114677	-707.094167	5.38	5.74
HT(c2-c2)-b	-707.058566	-707.037715	4.17	4.24	HT(c3-c3)-a	-707.114627	-707.094391	5.41	5.60
open(c2-c3)-a	-707.0582	-707.037316	4.40	4.49	open(c2-c3)-b	-707.114546	-707.094134	5.46	5.76
open(c2-c3)-c	-707.058094	-707.037161	4.47	4.59	open(c2-c3)-a	-707.114489	-707.094091	5.50	5.79
open(c2-c2)-c	-707.057923	-707.03704	4.57	4.67	HT(c2-c3)-d	-707.114213	-707.094027	5.67	5.83
open(c2-c2)-a	-707.057902	-707.036983	4.59	4.70	open(c2-c2)-c	-707.112878	-707.092618	6.51	6.71
open(c2-c2)-b	-707.057815	-707.036982	4.64	4.70	open(c2-c1)-a	-707.112843	-707.092213	6.53	6.97
open(c2-c3)-b	-707.057724	-707.037025	4.70	4.68	open(c2-c2)-a	-707.112457	-707.091973	6.78	7.12
open(c3-c2)-a	-707.057607	-707.036611	4.77	4.94	open(c2-c2)-b	-707.112347	-707.091919	6.84	7.15
open(c3-c2)-b	-707.057356	-707.036435	4.93	5.05	open(c3-c1)-a	-707.11225	-707.091723	6.91	7.27

Table S18. Stabilization of the dimeric conformers of **BzA-BH$_3$** through dihydrogen bonding (ΔE_{DHB} and ΔH_{DHB}) given in Hartree as well as their product with the respective Boltzmann weights ($\chi_{dimer}\cdot\Delta E_{DHB}$ and $\chi_{dimer}\cdot\Delta H_{DHB}$) given in kcal/mol. Calculations were carried out at B3LYP / 6-311++G(2d,p) / SMD(DCM) and B3LYP / 6-311++G(2d,p) / IEFPCM(DCM) with GD3BJ dispersion correction.

6-311++G(2d,p) / SMD(DCM)					6-311++G(2d,p) / IEFPCM(DCM) with GD3BJ				
**conf.**	ΔE_{DHB}	ΔH_{DHB}	$\chi_{dimer}\cdot\Delta E_{DHB}$	$\chi_{dimer}\cdot\Delta H_{DHB}$	**conf.**	ΔE_{DHB}	ΔH_{DHB}	$\chi_{dimer}\cdot\Delta E_{DHB}$	$\chi_{dimer}\cdot\Delta H_{DHB}$
HT(c1-c1)-a	-0.001972	-0.001087	-0.48	-0.28	HT(c1-c3)-c	-0.018432	-0.018199	-4.61	-4.79
HT(c1-c1)-b	-0.002019	-0.001062	-0.52	-0.26	HT(c1-c2)-d	-0.018503	-0.018242	-5.30	-5.28
open(c1-c1)-a	0.000007	0.001252	0.00	0.03	open(c1-c1)-a	-0.015697	-0.015400	-0.22	-0.21
HT(c1-c2)-b	-0.002780	-0.001768	-0.04	-0.02	open(c3-c1)-c	-0.015902	-0.015485	-0.29	-0.24
HT(c1-c2)-a	-0.002812	-0.001753	-0.04	-0.02	HT(c1-c3)-d	-0.015788	-0.015396	-0.25	-0.22
HT(c1-c3)-a	-0.002545	-0.001614	-0.02	-0.02	HT(c1-c2)-c	-0.013562	-0.013031	-0.25	-0.17
HT(c1-c2)-d	-0.002788	-0.001774	-0.03	-0.02	HT(c1-c1)-a	-0.012563	-0.012095	-0.08	-0.06
HT(c1-c3)-b	-0.002218	-0.001328	-0.02	-0.01	HT(c1-c1)-b	-0.012539	-0.012095	-0.08	-0.06
HT(c1-c3)-d	-0.002535	-0.001488	-0.02	-0.01	HT(c3-c3)-b	-0.016089	-0.015684	-0.03	-0.02
HT(c1-c3)-c	-0.002457	-0.001377	-0.02	-0.01	HT(c3-c3)-c	-0.016134	-0.015696	-0.03	-0.03
HT(c1-c2)-c	-0.002507	-0.001456	-0.02	-0.01	HT(c2-c2)-a	-0.016185	-0.015717	-0.03	-0.03
open(c2-c1)-a	-0.001629	-0.000381	-0.01	0.00	HT(c2-c3)-d	-0.015828	-0.015395	-0.02	-0.02
open(c3-c1)-c	-0.001185	0.000057	0.00	0.00	HT(c2-c3)-c	-0.015794	-0.015374	-0.02	-0.02
open(c3-c1)-a	-0.001190	0.000204	0.00	0.00	HT(c1-c2)-a	-0.012550	-0.012033	-0.01	0.00
open(c1-c2)-a	-0.003529	-0.002401	0.00	0.00	HT(c1-c3)-a	-0.012461	-0.011966	-0.01	0.00
open(c3-c1)-b	0.000072	0.001145	0.00	0.00	HT(c1-c3)-b	-0.012431	-0.011890	-0.01	0.00
HT(c3-c3)-a	-0.000018	0.001077	0.00	0.00	HT(c1-c2)-b	-0.012414	-0.011865	-0.01	0.00
HT(c2-c3)-a	-0.003363	-0.003319	0.00	-0.01	HT(c3-c3)-d	-0.014551	-0.013998	-0.01	0.00
HT(c3-c3)-b	-0.003092	-0.002134	0.00	0.00	HT(c2-c2)-b	-0.014524	-0.014010	0.00	0.00
HT(c3-c3)-c	-0.002918	-0.001923	0.00	0.00	open(c3-c1)-c	-0.011394	-0.010644	0.00	0.00
HT(c2-c2)-a	-0.003416	-0.002266	0.00	0.00	open(c1-c2)-a	-0.011450	-0.010691	0.00	0.00
HT(c2-c3)-b	-0.002869	-0.001969	0.00	0.00	open(c3-c2)-b	-0.012573	-0.011885	0.00	0.00
HT(c3-c3)-d	-0.003007	-0.002037	0.00	0.00	open(c3-c2)-a	-0.012580	-0.011898	0.00	0.00
HT(c2-c3)-c	-0.003167	-0.002090	0.00	0.00	HT(c2-c3)-a	-0.012247	-0.011579	0.00	0.00
HT(c2-c3)-d	-0.002832	-0.001747	0.00	0.00	open(c2-c3)-c	-0.012471	-0.011763	0.00	0.00
HT(c2-c2)-b	-0.002468	-0.001185	0.00	0.00	HT(c3-c3)-a	-0.012165	-0.011326	0.00	0.00
open(c2-c3)-a	-0.001716	-0.000563	0.00	0.00	open(c2-c3)-b	-0.012222	-0.011369	0.00	0.00
open(c2-c3)-c	-0.002192	-0.000854	0.00	0.00	open(c2-c3)-a	-0.011889	-0.011262	0.00	0.00
open(c2-c2)-c	-0.002086	-0.000699	0.00	0.00	HT(c2-c3)-d	-0.012353	-0.011402	0.00	0.00
open(c2-c2)-a	-0.001717	-0.000452	0.00	0.00	open(c2-c2)-c	-0.010610	-0.009900	0.00	0.00
open(c2-c2)-b	-0.001804	-0.000453	0.00	0.00	open(c2-c1)-a	-0.008148	-0.007189	0.00	0.00
open(c2-c3)-b	-0.001825	-0.000510	0.00	0.00	open(c2-c2)-a	-0.010079	-0.009201	0.00	0.00
open(c3-c2)-a	-0.001599	-0.000149	0.00	0.00	open(c2-c2)-b	-0.010189	-0.009255	0.00	0.00
open(c3-c2)-b	-0.001348	0.000027	0.00	0.00	open(c3-c1)-a	-0.007499	-0.006652	0.00	0.00
	$\sum(\chi_{dimer}\cdot\Delta E_{DHB}/\Delta H_{DHB})$ =		-1.24	-0.67		$\sum(\chi_{dimer}\cdot\Delta E_{DHB}/\Delta H_{DHB})$ =		-11.26	-11.16

Table S19. Conformers of monomeric (*S,S*)-**Bis(MBA)-BH$_3$** and its corresponding relative zero-point corrected energies (ΔE_{ZPC}), enthalpies (ΔH) and Gibbs free energies (ΔG_{298K}) given in kcal/mol. The Boltzmann weights χ are given in percentage. The angles α = C_{Ar}-C^{α}-N-B and β = C_{Ar}-C_{Ar}-C^{α}-N are given in degree. Calculations were carried out at B3LYP / 6-311++G(2d,p) / IEFPCM(DCM).

conf.	α-1	β-1	α-2	β-2	ΔE_{ZPC}	ΔH	ΔG_{298K}	$\chi(\Delta E_{ZPC})$	$\chi(\Delta H)$	$\chi(\Delta G_{298K})$
c1	58.3	-78.5	168.6	-53.9	0.00 [a)]	0.00 [b)]	0.08	50.7	52.9	45.5
c2	88.1	-92.3	70.5	106.7	0.05	0.11	0.00 [c)]	46.4	44.2	52.0
c3	81.4	-14.6	-63.1	-64.3	1.71	1.73	1.81	2.8	2.8	2.4
c4	26.1	-86.3	-82.7	-102.3	3.19	3.21	3.28	0.0	0.0	0.0
c5	91.8	-85.2	-152.5	76.6	4.29	4.25	4.76	0.0	0.0	0.0

[a)] referenced to E_{ZPC}(c1) = -702.420313 hartree.
[b)] referenced to H(c1) = -702.402286 hartree.
[c)] referenced to G_{298K}(c2) = -702.465981 hartree.

Table S20. Conformers of dimeric (*S*,*S*)-**Bis(MBA)-BH₃** and its corresponding relative zero-point corrected energies (ΔE_{ZPC}), enthalpies (ΔH) and Gibbs free energies (ΔG_{298K}) given in kcal/mol. The Boltzmann weights χ are given in percentage. Calculations were carried out at B3LYP / 6-311++G(2d,p) / IEFPCM(DCM) for dimer analysis.

conf.	ΔE_{ZPC}	ΔH	ΔG_{298K}	$\chi(\Delta E_{ZPC})$	$\chi(\Delta H)$	$\chi(\Delta G_{298K})$
HT(c1-c2)	0.00[a)]	0.00[b)]	0.56	56.6	62.4	13.2
HT(c1-c1)	0.57	0.68	0.18	21.4	19.6	25.1
open(c1-c1)	0.98	1.17	0.12	10.7	8.7	27.7
open(c1-c2)	0.96	1.13	0.00[c)]	11.2	9.3	34.0

[a)] referenced to E_{ZPC}(HT(c1-c2)) = -1404.84286 hartree.
[b)] referenced to H(HT(c1-c2)) = -1404.80562 hartree.
[c)] referenced to G_{298K}(open(c1-c2)) = -1404.91687 hartree.

Table S21. Conformer energies (E_{ZPC}) and enthalpies (H) of monomeric and dimeric **Bis(MBA)-BH₃** and their corresponding calculated stabilization through dihydrogen bonding (ΔE_{DHB} and ΔH_{DHB}) given in Hartree as well as their product with the respective Boltzmann weights ($\chi_{dimer}\cdot\Delta E_{DHB}$ and $\chi_{dimer}\cdot\Delta H_{DHB}$) in Hartree and kcal/mol. Calculations were carried out at B3LYP / 6-311++G(2d,p) / IEFPCM(DCM).

conf.	E	H	ΔE_{DHB}	ΔH_{DHB}	$\chi_{dimer}\cdot\Delta E_{DHB}$	$\chi_{dimer}\cdot\Delta H_{DHB}$	$\chi_{dimer}\cdot\Delta E_{DHB}$	$\chi_{dimer}\cdot\Delta H_{DHB}$
c1	-702.420313	-702.402286						
c2	-702.42023	-702.402116						
c3	-702.417594	-702.399522						
c4	-702.415236	-702.397168						
c5	-702.413479	-702.395507						
HT(c1-c1)-a	-1404.842855	-1404.805621	-0.002229	-0.0010	-0.001261	-0.000654	-0.79	-0.41
HT(c1-c1)-b	-1404.84194	-1404.804531	-0.001397	-0.0001	-0.000300	-0.000025	-0.19	-0.02
HT(c1-c2)-a	-1404.841288	-1404.803758	-0.000745	0.0006	-0.000080	0.000056	-0.05	0.04
HT(c1-c2)-b	-1404.841331	-1404.803828	-0.000705	0.0007	-0.000079	0.000069	-0.05	0.04
				$\sum$ ($\chi_{dimer}\cdot\Delta E_{DHB}/\Delta H_{DHB}$) =	-0.001720	-0.000554	-1.08	-0.35

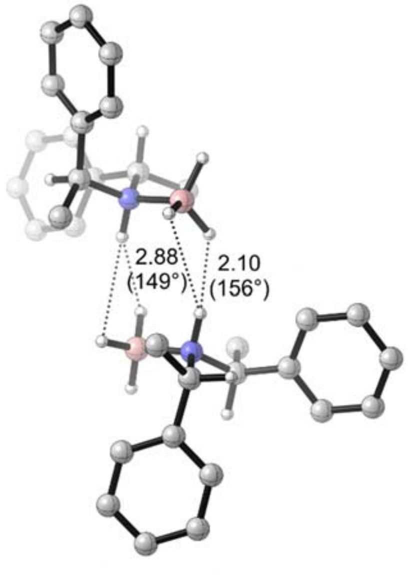

Fig. S25. Structure of the (*S*,*S*)-**Bis(MBA)-BH₃** dimer.

8. Crystallography

The crystal structure was analysed on a Rigaku Synergy dual source device, with Cu micro focus sealed tube (Cu Kα) using mirror monochromators and a HyPix-6000HE: Hybrid photon counting X-ray detector. The crystal was mounted in a Hampton CryoLoops using GE/Bayer silicone grease. Data was recorded and reduced using the CrysalisPro Software.[2] The structure was solved using WinGX[3] in combination with ShelXT[4] and refined with shelXle[5] and ShelXL.

Table S22. Crystal data and structure refinement for (N*R*,α*R*)-**DMBA-BH$_3$**.

CCDC-Nr	2205463
Empirical formula	C9 H16 B N
Formula weight [g/mol]	149.04
Crystal system	Monoclinic
Space group	$P2_1$ (4)
Lattice parameters [Å]	
a	9.98504(17)
b	10.23444(16)
c	10.4134(2)
α	90
β	111.278(2)
γ	90
Density [g/cm^3]	0.998
Crystal size [mm^3]	0.323 x 0.199 x 0.129
Volume [Å^3]	991.62(3)
Z	4
Temperature [K]	169.98(10)
Diffraction Device	XtaLAB Synergy, Dualflex, HyPix
Radiation Type	1.54184 Å (Cu K/ micro-focus sealed X-ray tube)
F(000)	328
Absorption coefficient [mm^{-1}]	0.417
Absorption correction	Gaussian
Measurement range	4.6 - 66.5
Index range	-11 < h < 11
	-12 < k < 12
	-12 < l < 11
Measured reflexes	9255
Independent	3321
Observed	3178
R(int)	0.0329
Completeness (%) / theta (°)	100.0 / 66.469
Transmission (min / max)	0.483 / 1.000
R1 (observed/all)	0.0341 / 0.0356
wR2 (observed/all)	0.0881 / 0.0895
GooF = S	1.041
Rest electron density max./min. [e-/Å^3]	-0.143 / 0.094

Table S23. Atomic coordinates and equivalent isotropic displacement parameters [Å^2] for (N*R*,α*R*)-**DMBA-BH$_3$**

	x	y	z	U(eq)
N(1)	0.39890(15)	0.55231(15)	0.07180(15)	0.0327(3)
C(1)	0.1085(2)	0.6107(2)	0.0946(2)	0.0424(4)
C(2)	-0.0397(2)	0.6077(2)	0.0591(2)	0.0466(5)
C(3)	-0.1277(2)	0.6747(2)	-0.0554(2)	0.0465(5)
C(4)	-0.0684(2)	0.7422(2)	-0.1356(2)	0.0498(5)
C(5)	0.0791(2)	0.7443(2)	-0.1015(2)	0.0425(4)
C(6)	0.16944(18)	0.67885(18)	0.01425(18)	0.0337(4)
C(7)	0.33126(19)	0.68585(18)	0.05226(18)	0.0341(4)
C(8)	0.4002(2)	0.7702(2)	0.1800(2)	0.0451(5)
C(9)	0.3254(2)	0.4664(2)	-0.0486(2)	0.0431(4)
B(1)	0.5694(2)	0.5544(3)	0.1015(2)	0.0421(5)
N(2)	0.62583(16)	0.36674(17)	0.38731(17)	0.0381(4)
C(11)	0.88514(19)	0.39854(19)	0.53262(19)	0.0367(4)
C(12)	0.9317(2)	0.4636(2)	0.4400(2)	0.0462(5)
C(13)	1.0739(2)	0.4560(3)	0.4514(2)	0.0538(6)
C(14)	1.1714(2)	0.3845(3)	0.5553(2)	0.0535(6)
C(15)	1.1263(2)	0.3202(2)	0.6486(2)	0.0539(6)
C(16)	0.9841(2)	0.3265(2)	0.6374(2)	0.0461(5)
C(17)	0.7309(2)	0.4086(2)	0.52568(19)	0.0407(5)
C(18)	0.6983(3)	0.5446(3)	0.5613(3)	0.0644(7)
C(19)	0.6552(2)	0.2302(2)	0.3564(2)	0.0509(5)
B(2)	0.4582(2)	0.3821(3)	0.3686(3)	0.0480(6)
HN1A	0.382(2)	0.517(2)	0.145(2)	0.031(5)
H(1)	0.1686	0.565762	0.174567	0.051
H(2)	-0.080533	0.559453	0.113754	0.056
H(3)	-0.228947	0.674154	-0.078689	0.056
H(4)	-0.128958	0.787634	-0.214983	0.06
H(5)	0.118898	0.791155	-0.157949	0.051
H(7)	0.34984	0.728819	-0.025909	0.041
H(8A)	0.393096	0.725649	0.260553	0.068
H(8B)	0.350264	0.85438	0.167253	0.068
H(8C)	0.501607	0.784833	0.194224	0.068
H(9A)	0.222408	0.462802	-0.064801	0.065
H(9B)	0.366089	0.378195	-0.029709	0.065
H(9C)	0.339151	0.501539	-0.130421	0.065
HB1A	0.584(3)	0.609(3)	0.015(3)	0.064(8)
HB1B	0.602(2)	0.451(3)	0.104(2)	0.047(6)
HB1C	0.625(2)	0.597(2)	0.205(2)	0.038(5)
HN2A	0.639(2)	0.416(2)	0.321(2)	0.037(5)
H(12)	0.865477	0.513792	0.368048	0.055
H(13)	1.104391	0.500674	0.386783	0.065
H(14)	1.268958	0.379532	0.562685	0.064
H(15)	1.193259	0.27116	0.721074	0.065

H(16)	0.953984	0.281285	0.701831	0.055
H(17)	0.719923	0.347718	0.596355	0.049
H(18A)	0.771575	0.571412	0.649044	0.097
H(18B)	0.603577	0.54567	0.569473	0.097
H(18C)	0.69853	0.605218	0.48852	0.097
H(19A)	0.638259	0.170741	0.422703	0.076
H(19B)	0.75546	0.222821	0.363328	0.076
H(19C)	0.591341	0.20711	0.262911	0.076
HB2A	0.434(2)	0.486(3)	0.369(2)	0.049(6)
HB2B	0.448(3)	0.326(3)	0.455(3)	0.058(7)
HB2C	0.397(3)	0.340(3)	0.264(2)	0.051(6)

Table S24. Anisotropic displacement parameters [Å^2] for (N*R*,α*R*)-**DMBA-BH$_3$**

	U^{11}	U^{22}	U^{33}	U^{23}	U^{13}	U^{12}
N(1)	0.0290(7)	0.0369(8)	0.0331(7)	0.0001(6)	0.0125(6)	-0.0016(6)
C(1)	0.0372(9)	0.0528(12)	0.0406(10)	0.0078(9)	0.0181(8)	0.0034(9)
C(2)	0.0411(10)	0.0528(12)	0.0537(11)	0.0015(10)	0.0265(9)	-0.0025(9)
C(3)	0.0304(9)	0.0504(12)	0.0588(12)	-0.0079(10)	0.0164(9)	0.0011(9)
C(4)	0.0385(10)	0.0505(13)	0.0552(12)	0.0093(10)	0.0106(9)	0.0091(9)
C(5)	0.0401(10)	0.0398(11)	0.0487(11)	0.0082(8)	0.0176(8)	0.0040(8)
C(6)	0.0322(9)	0.0338(9)	0.0376(9)	-0.0019(8)	0.0156(7)	0.0011(7)
C(7)	0.0332(9)	0.0347(9)	0.0368(9)	0.0006(7)	0.0157(7)	-0.0014(7)
C(8)	0.0400(10)	0.0439(11)	0.0517(11)	-0.0112(9)	0.0172(9)	-0.0059(9)
C(9)	0.0392(9)	0.0430(11)	0.0461(10)	-0.0108(9)	0.0141(8)	-0.0012(8)
B(1)	0.0287(10)	0.0510(13)	0.0482(13)	0.0063(11)	0.0157(9)	0.0019(10)
N(2)	0.0315(8)	0.0446(9)	0.0390(8)	0.0047(7)	0.0139(7)	-0.0019(7)
C(11)	0.0325(9)	0.0381(10)	0.0369(9)	-0.0001(8)	0.0095(7)	0.0006(8)
C(12)	0.0341(9)	0.0571(13)	0.0455(10)	0.0095(10)	0.0120(8)	0.0003(9)
C(13)	0.0404(10)	0.0719(15)	0.0524(12)	-0.0015(11)	0.0208(9)	-0.0081(11)
C(14)	0.0286(9)	0.0597(14)	0.0690(13)	-0.0185(12)	0.0138(9)	-0.0005(9)
C(15)	0.0367(10)	0.0445(12)	0.0634(13)	-0.0042(10)	-0.0022(10)	0.0067(9)
C(16)	0.0422(11)	0.0437(11)	0.0451(11)	0.0061(9)	0.0070(8)	0.0006(9)
C(17)	0.0344(9)	0.0530(12)	0.0363(9)	0.0025(8)	0.0149(8)	0.0010(8)
C(18)	0.0442(11)	0.0741(17)	0.0740(16)	-0.0279(14)	0.0203(11)	0.0064(11)
C(19)	0.0461(11)	0.0440(11)	0.0624(13)	-0.0079(10)	0.0196(10)	-0.0043(9)
B(2)	0.0299(10)	0.0605(16)	0.0551(14)	0.0134(12)	0.0173(10)	-0.0017(10)

9. NMR spectra

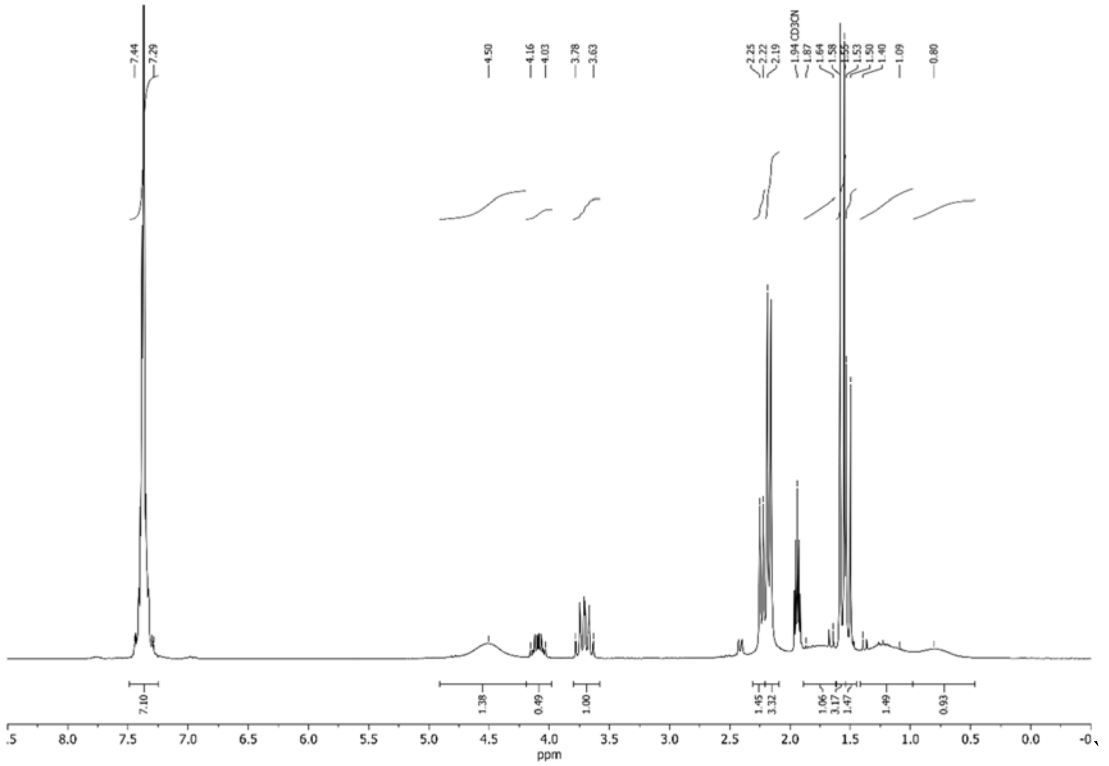

Fig. S26. 200 MHz ^{1}H-NMR diastereomeric mixture of (N*R*)/(N*S*)-**DMBA-BH$_3$** in ACN-*d_3*

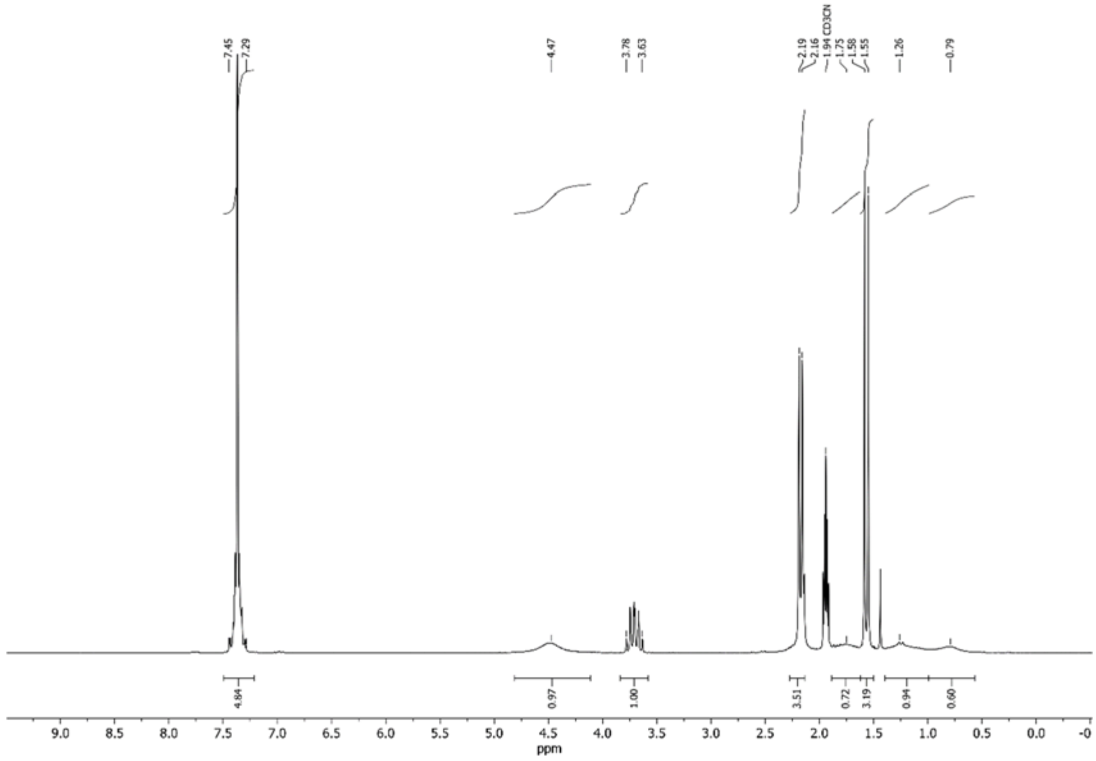

Fig. S27. 200 MHz ^{1}H-NMR of the major species (N*R*,α*R*)/(N*S*,α*S*)-**DMBA-BH$_3$** in ACN-*d_3*

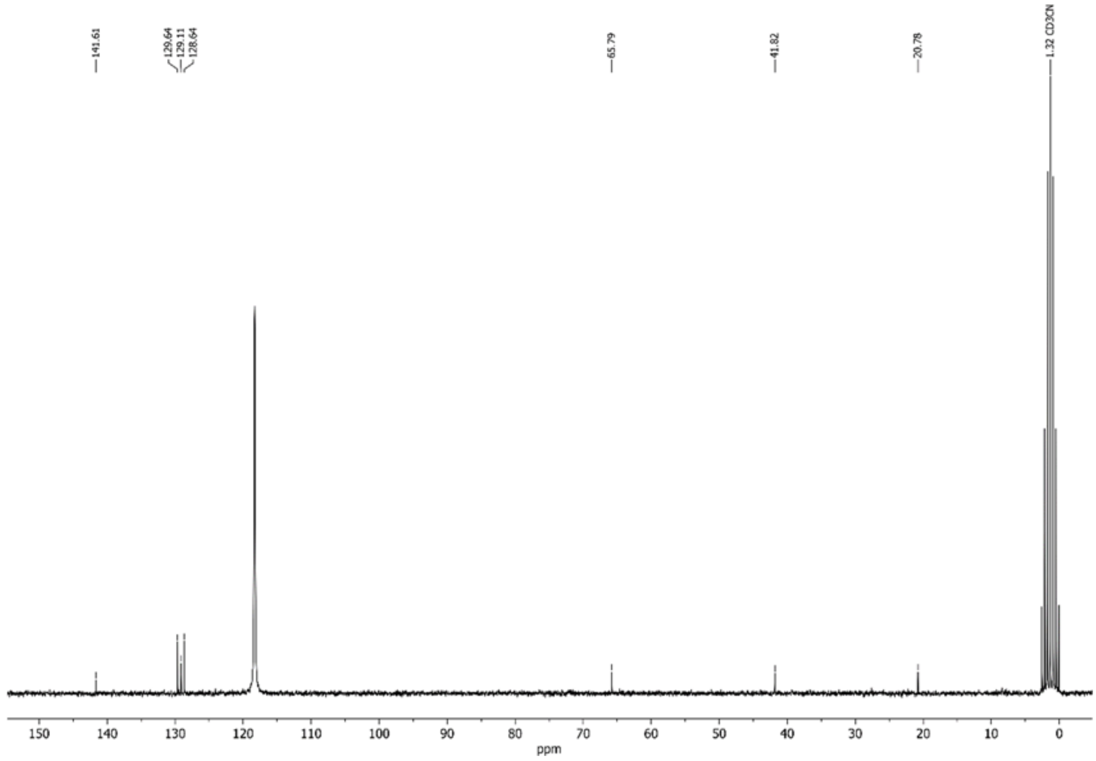

Fig. S28. 50 MHz ^{13}C-NMR of the major species (N*R*,α*R*)/(N*S*,α*S*)-**DMBA-BH$_3$** in ACN-d_3

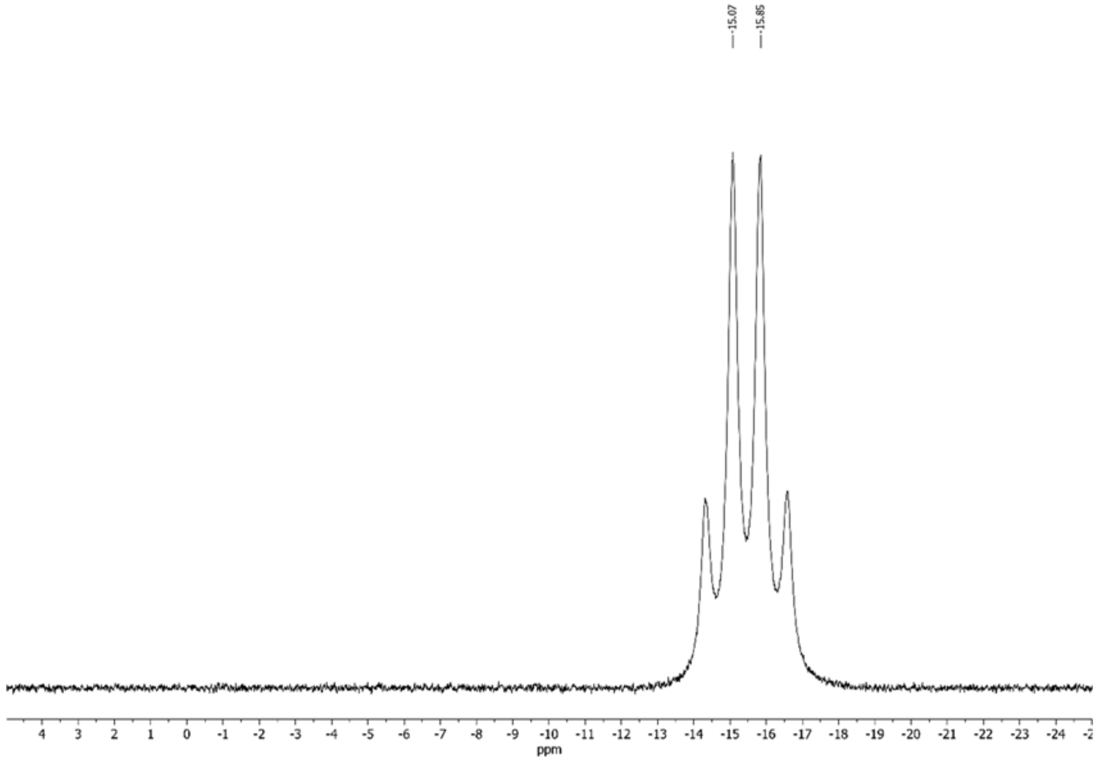

Fig. S29. 128 MHz ^{11}B-NMR of the major species (N*R*,α*R*)/(N*S*,α*S*)-**DMBA-BH$_3$** in ACN-d_3

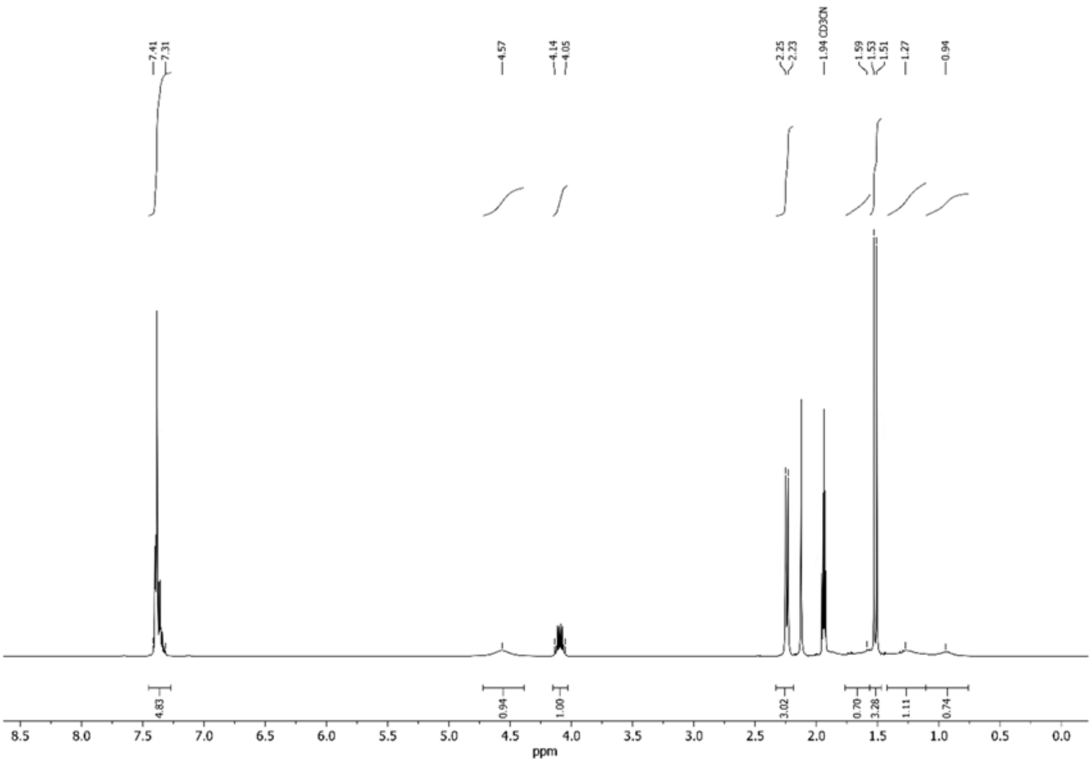

Fig. S30. 200 MHz ^{1}H-NMR of the minor species (N*S*,α*R*)/(N*R*,α*S*)-**DMBA-BH₃** in ACN-*d₃*

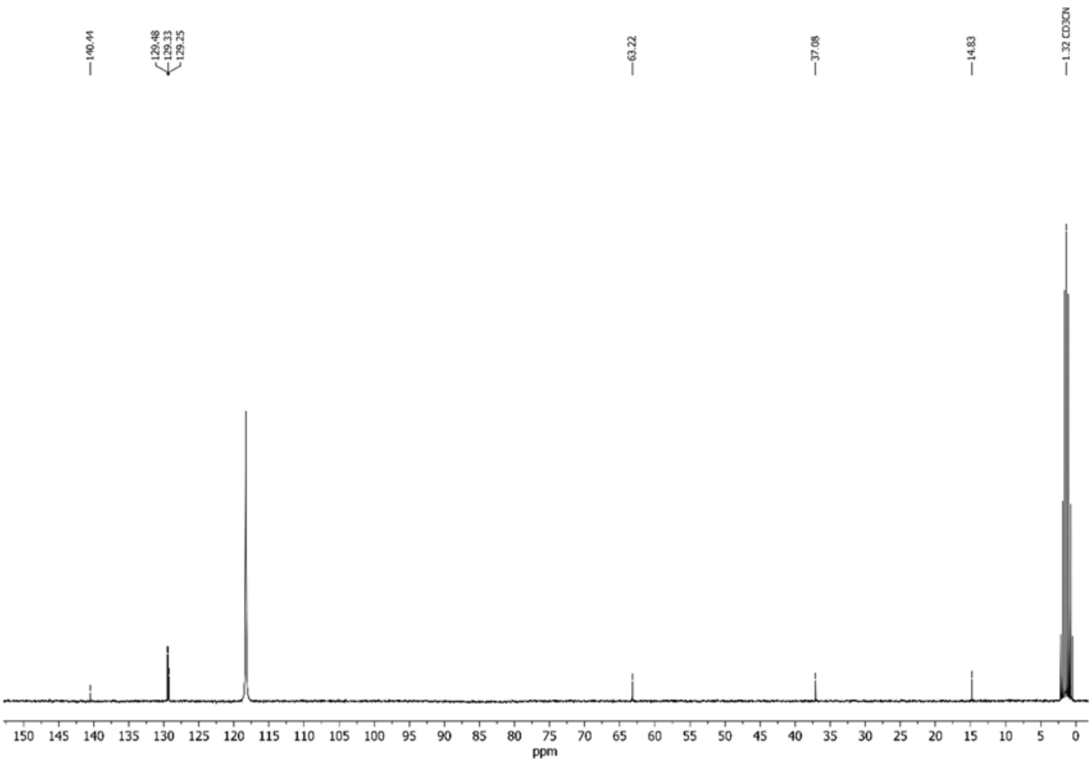

Fig. S31. 50 MHz ^{13}C-NMR of the minor species (N*S*,α*R*)/(N*R*,α*S*)-**DMBA-BH₃** in ACN-*d₃*

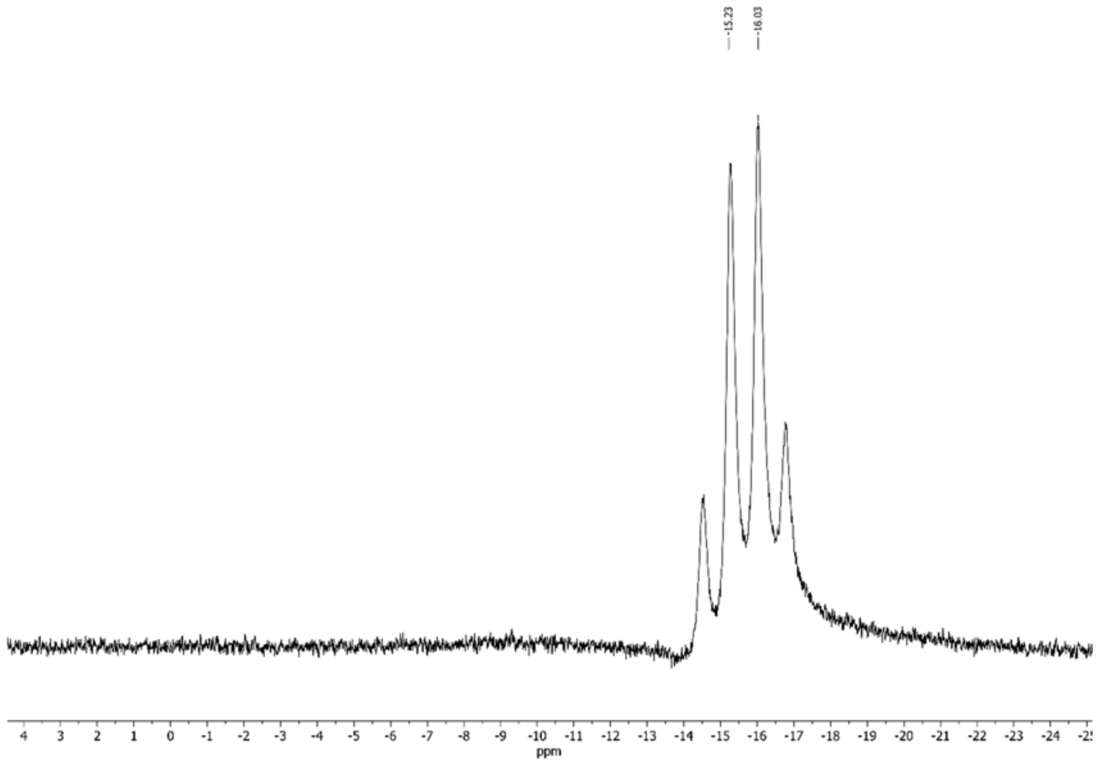

Fig. S32. 128 MHz ^{11}B-NMR of the minor species (N*S*,α*R*)/(N*R*,α*S*)-**DMBA-BH$_3$** in ACN-d_3

10. References

1. *Gaussian 09, Rev E.01*, M. J. Frisch, G. W. Trucks, H. B. Schlegel, G. E. Scuseria, M. A. Robb, J. R. Cheeseman, G. Scalmani, V. Barone, B. Mennucci, G. A. Petersson, H. Nakatsuji, M. Caricato, X. Li, H. P. Hratchian, A. F. Izmaylov, J. Bloino, G. Zheng, J. L. Sonnenberg, M. Hada, M. Ehara, K. Toyota, R. Fukuda, J. Hasegawa, M. Ishida, T. Nakajima, Y. Honda, O. Kitao, H. Nakai, T. Vreven, J. J. A. Montgomery, J. E. Peralta, F. Ogliaro, M. Bearpark, J. J. Heyd, E. Brothers, K. N. Kudin, V. N. Staroverov, T. Keith, R. Kobayashi, J. Normand, K. Raghavachari, A. Rendell, J. C. Burant, S. S. Iyengar, J. Tomasi, M. Cossi, N. Rega, J. M. Millam, M. Klene, J. E. Knox, J. B. Cross, V. Bakken, C. Adamo, J. Jaramillo, R. Gomperts, R. E. Stratmann, O. Yazyev, A. J. Austin, R. Cammi, C. Pomelli, J. W. Ochterski, R. L. Martin, K. Morokuma, V. G. Zakrzewski, G. A. Voth, P. Salvador, J. J. Dannenberg, S. Dapprich, A. D. Daniels, O. Farkas, J. B. Foresman, J. V. Ortiz, J. Cioslowski and D. J. Fox, Wallingford CT, USA, 2013

2. Meyer, M., Paciorek, W., Kowalski, A., Muszynski, A., Wisniewski, A., Pol, M., Przewozniczek, M., Stec, P., Bujnik, D., Kulza, H., Grzesczyk, M., Kuna, T., Serwata, L., Prochniak, G., Piotrowski, W., Antkowiak, M., Jasiak, R., Idzi, M., Yoshida, H., Hendrixson, T., Archeson, C. & Wodziczko, T. (2018). CrysAlisPro: Rigaku Oxford Diffraction (1995-2018).

3. Farrugia, L. J. WinGX suite for small-molecule single-crystal crystallography. J. Appl. Crystallogr. 1999, 32, 837–838, DOI: 10.1107/S0021889899006020.

4. Sheldrick, G. M. A short history of SHELX. Acta Crystallogr A Found Crystallogr 2008, 64, 112–122, DOI: 10.1107/S0108767307043930.

5. Hübschle, C. B.; Sheldrick, G. M.; Dittrich, B. ShelXle: a Qt graphical user interface for SHELXL. J. Appl. Crystallogr. 2011, 44, 1281–1284, DOI: 10.1107/S0021889811043202. 5 Putz, H.; Brandenburg, K. Diamond; Crystal Impact: Bonn, Deutschland, 2014
